# Human Serum Albumin Labelling with a New BODIPY Dye Having a Large Stokes Shift

**DOI:** 10.3390/molecules26092679

**Published:** 2021-05-03

**Authors:** Valeria I. Raskolupova, Tatyana V. Popova, Olga D. Zakharova, Anastasia E. Nikotina, Tatyana V. Abramova, Vladimir N. Silnikov

**Affiliations:** 1 Institute of Chemical Biology and Fundamental Medicine SB RAS, Lavrent’ev Ave, 8, 630090 Novosibirsk, Russia; v.raskolupova@mail.ru (V.I.R.); popovatv@niboch.nsc.ru (T.V.P.); isar@niboch.nsc.ru (O.D.Z.); a.nikotina@mail.ru (A.E.N.); silnik@niboch.nsc.ru (V.N.S.); 2 Faculty of Natural Sciences, Novosibirsk State University, Pirogova St., 2, 630090 Novosibirsk, Russia

**Keywords:** BODIPY dye with a large stokes shift, *N*-trifluoroacetylhomocysteine thiolactone, biopolymer labelling, HSA, theranostic

## Abstract

BODIPY dyes are photostable neutral derivatives of 4,4-difluoro-4-bora-3a,4a-diaza-*s*-indacene. These are widely used as chemosensors, laser materials, and molecular probes. At the same time, BODIPY dyes have small or moderate Stokes shifts like most other fluorophores. Large Stokes shifts are preferred for fluorophores because of higher sensitivity of such probes and sensors. The new boron containing BODIPY dye was designed and synthesized. We succeeded to perform an annulation of pyrrole ring with coumarin heterocyclic system and achieved a remarkable difference in absorption and emission maximum of obtained fluorophore up to 100 nm. This BODIPY dye was equipped with linker arm and was functionalized with a maleimide residue specifically reactive towards thiol groups of proteins. BODIPY residue equipped with a suitable targeting protein core can be used as a suitable imaging probe and agent for Boron Neutron Capture Therapy (BNCT). As the most abundant protein with a variety of physiological functions, human serum albumin (HSA) has been used extensively for the delivery and improvement of therapeutic molecules. Thiolactone chemistry provides a powerful tool to prepare albumin-based multimodal constructions. The released sulfhydryl groups of the homocysteine functional handle in thiolactone modified HSA were labeled with BODIPY dye to prepare a labeled albumin-BODIPY dye conjugate confirmed by MALDI-TOF-MS, UV-vis, and fluorescent emission spectra. Cytotoxicity of the resulting conjugate was investigated. This study is the basis for a novel BODIPY dye-albumin theranostic for BNCT. The results provide further impetus to develop derivatives of HSA for delivery of boron to cancer cells.

## 1. Introduction

Boron neutron capture therapy (BNCT) is a promising cancer treatment modality based on the nuclear capture of slow neutrons by stable ^10^B atoms followed by charged particle emission that have a cell killing effect within a 10-μm range [1]. Successful BNCT mainly depends on the selective accumulation of ^10^B including agents in tumor cells. Nowadays, only two boron agents, boronophenylalanine (BPA) and borocaptate sodium (BSH), have been used clinically [2,3,4]. However, both compounds do not meet all the required criteria. Today, third-generation boron-containing drugs for BNCT (water-soluble boron-containing constructs capable of selectively delivering stable ^10^B isotope to tumor cells at a 20 to 50 μg of ^10^B per gram of tumor) are being developed [5,6]. 

Due to continuous blood circulation and low immunological effect, human serum albumin (HSA) is successfully used as a core to improve the potential of therapeutic agents in theranostic constructions [7,8,9,10,11,12,13,14,15]. The efficiency of HSA as a carrier of cytostatics has been proven [16,17,18]. However, there is a problem not only in the selective accumulation of boron in a tumor for BNCT therapy [19], but also in the quantitative estimation of its accumulation. Therefore, theranostics may contain fluorescence residues for sensing [20].

Fluorescent labeling is one of the best available methods for sensing. BODIPY dyes are fluorescent compounds that have many advantages over other classes of dyes, such as high extinction, high fluorescent quantum yields, and excellent photo and chemical stability [21]. However, most BODIPY dyes have small or moderate Stokes shifts (15–70 nm) [21,22,23,24]. The annulation of 7-diethylaminocoumarin to the BODIPY core resulted in increasing the Stocks shift and shifting an emission to near infrared region (NIR) [25]. A NIR emission better penetrates into tissues than the shorter wavelength light avoiding an interaction with cell components, so near-infrared radiation is harmless to living cells [23,24]. The last is important in the case of BODIPY-based theranostic creation. 

The binding of BODIPY residues to proteins is a rather recent topic for research. BODIPY residues were used as pH-activable fluorescent imaging probe for cancer diagnosis. Novel acidic probes based on the BODIPY fluorophore were synthesized, and then covalently conjugated to a cancer-targeting monoclonal antibody. As proof of concept, ex and in vivo imaging of HER2-positive lung cancer cells in mice were performed [26]. Nanocomplex of nanoparticles from hydrophobic 4,4-difluoro-4-bora-3a,4a-diaza-s-indacene (BODIPY)-containing conjugated polymers and HSA was constructed. It exhibits robust stability in physiological conditions, excellent photothermic activity upon irradiation, the benefited accumulation in tumour, and optimal timing of treatment [27]. The pH probe, which has two xanthene donors and one BODIPY acceptor, was designed. This probe was used to image a non-covalent conjugate of the probe with bovine serum albumin (BSA) that was imported into endosomes or in the cytosol [28]. Authors of another recent work investigated the molecular interactions of two 2,6-diiodo-BODIPY derivatives with HSA using combined experimental and computational techniques [29]. It should be noted that functionalization of the BODIPY core for further covalent attachment to biopolymers is not a trivial task and often requires sophisticated synthetic procedures and reagents, or is not applicable in the synthesis of non-symmetrical BODIPY core [30,31,32,33]. 

Several publications describe the synthesis of covalent BODIPY conjugates of bovine serum albumin (BSA) used as a model protein. It should be mentioned that BSA, in contrast to HSA, is rather a model protein in such studies, while HSA is more suitable for creating theranostics. In the publication [34], the isothiocyanate method of bioconjugation was used with a large excess of the dye to the protein. However, it has been shown [12] that the sites of effective attachment to albumin can influence the functioning of albumin-based drugs. So, it should be taken into account when designing the conjugates with desirable pharmaceutical properties. The most successful protocol for purification of the protein conjugate described in [34] includes centrifugal dialysis against 70% ethanol. Serum albumin is a fairly stable protein, but it is known that a high ratio of organic solvent in aqueous solutions (more than 30% of ethanol or more than 20% of dimethyl sulfoxide (DMSO) destabilize the native structure of this protein [35,36]. This may lead to a change of biochemical properties of labelled albumin after renaturation. Therefore, the purification of labeled HSA by a centrifugal dialysis against a solution with a high organic solvent content seems not suitable. Also, the authors of [34] detected a fluorescence decrease of the BODIPY dye after bioconjugation. The authors explained this by the formation of thiourea bond in the course of covalent conjugation.

In the work [37], bioconjugation of BODIPY to BSA was carried out by Cu(I) catalyzed azide-alkyne cycloaddition via tyrosine residues. In this work, a large excess of the dye was used to achieve a high loading of the dye at the protein. The BSA contains 21 tyrosine residues, and the authors achieved BODIPY loading ca.1.4/1 (fluorophore/protein). However, this approach does not provide the selective labelling of a definite tyrosine residue and may result in structural changes at protein sites important for the interaction with cell receptors. Bioconjugation of BODIPY with BSA was carried out also via amide bond formation or thiol-ene conjugation with maleimide derivative of BODIPY [38]. The protein loading achieved through the carbodiimide method was 1–2 BODIPY dye per protein molecule. Loading obtained through thiol-ene conjugation has not been determined, but has been proven by high pressure liquid chromatography (HPLC) method. It should be noted, that Stokes shifts of BODIPY-BSA conjugates in [34,37,38] were not more than 75 nm, and in most cases they were about 30–50 nm.

Albumin molecule has 35 cysteine residues, and the Cys 34, the only one free cysteine, can react with maleimide moiety [7]. Most commercial HSA preparations have approximately 30–40% Cys 34 in a free and non-oxidized form [39,40]. Thus, conjugation of any molecule to Cys 34 has both positive and negative aspects. The positive aspect is the selective modification, which preserves biochemical properties of the resulting albumin conjugate. The negative aspect is extremely low protein loading. The consequences of the latter aspect can be overcome by increasing the amount of free thiol groups in the protein structure. However, the reduction of disulfide bridges of albumin can lead to the loss of the biochemical properties. Therefore, the selective modification of albumin with reagents capable of increasing the number of free thiol groups is relevant. 

Herein, we report a synthesis and a spectral study of a new non-symmetric BODIPY dye having 7-dimethylaminochromeno[4,3-b]pyrrol-4-one and pyrrole equipped with a linker arm carrying a carboxyl group for biopolymer labelling. We used HSA as a core for perspective theranostic molecule, and *N*-trifluoroacetylhomocysteine thiolactone as a selective protein modifier that allows introducing several additional free thiol groups into protein for thiol-“click” passage. Conjugation of this new dye to *N*-trifluoroacetylhomocysteinylated HSA and some physico-chemical properties of the new covalent conjugate are described. 

## 2. Results and Discussion

### 2.1. Chemistry

**Scheme 1:**
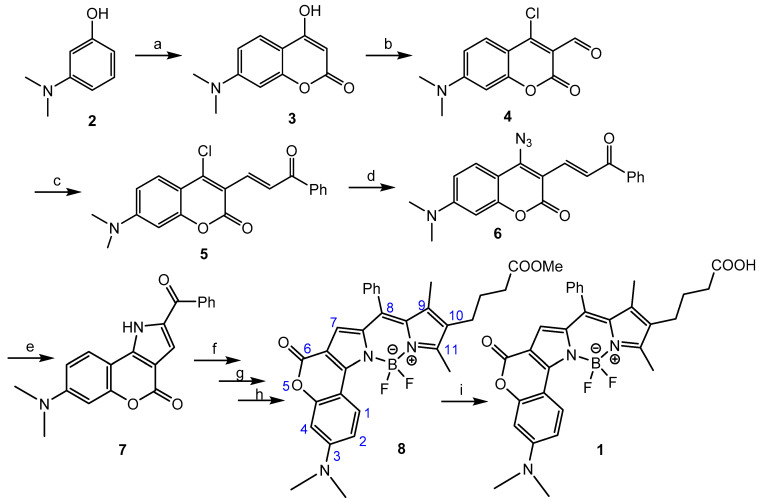
Synthesis of BODIPY **1**. Conditions: a—CH_2_(COOPh)_2_, toluene, reflux, 10 h, yield 89%; b—POCl_3_, DMF, RT, 24 h, yield 82%; c—Ph_3_P = CHBz, CH_2_Cl_2_, RT, 24 h, yield 35%; d—NaN_3_, acetone, 40 °C, 2 h; e–toluene, reflux, 2 h; f—POCl_3_, CH_2_Cl_2_, RT 48 h; g–**10**, POCl_3_, CH_2_Cl_2_, RT, 96 h; h—TEA (5 eq), BF_3_•OEt_2_ (30 eq), RT, 48 h, yield 89%; i—conc. aq. HCl:1,4-dioxane 1:5, RT, 6 h, quant.

A condensation of α-proton containing pyrrole with α-ketopyrrole was described as a direct and convenient method to achieve asymmetrically substituted BODIPY dyes [21]. The synthetic route to the target coumarin-fused BODIPY **1** is shown in Scheme 1. 

**Scheme 2:**
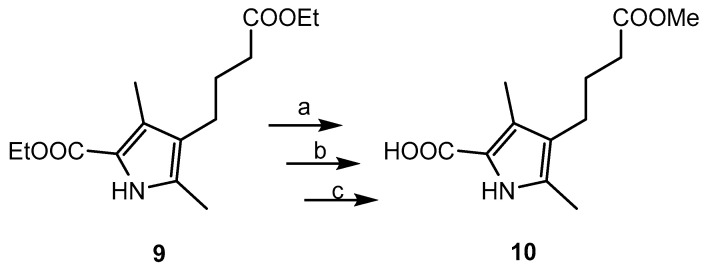
Synthesis of pyrrole-2-carboxylic acid **10**. Conditions: a—NaOBn, BnOH, 180 °C, 2 h, yield 50%; b—H_2_SO_4_, MeOH, RT, 2 h, yield 38%; c—H_2_, Pd/C, RT, 1 h, quant.

First, 3-(dimethylamino)phenol **2** was coupled with diphenyl malonate to give hydroxycoumarin **3** (Scheme 1, step a) [41]. This was then recrystallized from ethanol and used for four-step synthesis of BODIPY precursor **7** according to the procedure reported by Bochkov et al. [25] without significant modifications (7-dimethylaminocoumarin was used instead of 7-diethylaminocoumarin, see Supplementary Materials for synthetic protocols and physico-chemical characteristics of compounds **3**–**7**). Preparation of some BODIPY dyes by condensation with pyrrole dicarboxylates was reported in the literature [42]. So, we synthesized pyrrole diester **9** (Scheme 2) by a modified Paal-Knorr reaction [43]. Both carboxyl protective groups were removed from diester **9** as per the previous protocols [42,44], but the precipitation of pyrrole dicarboxylic acid under acidic conditions was not successful in our hands. 

We also failed to isolate the intermediate diacid using 1 M aqueous triethylammonium bicarbonate, pH 7.5, (TEAB) treatment. So, we applied protocol [45] and obtained the monoester derivative **10** from known compound **9** in three steps (Scheme 2).

**Figure 1:**
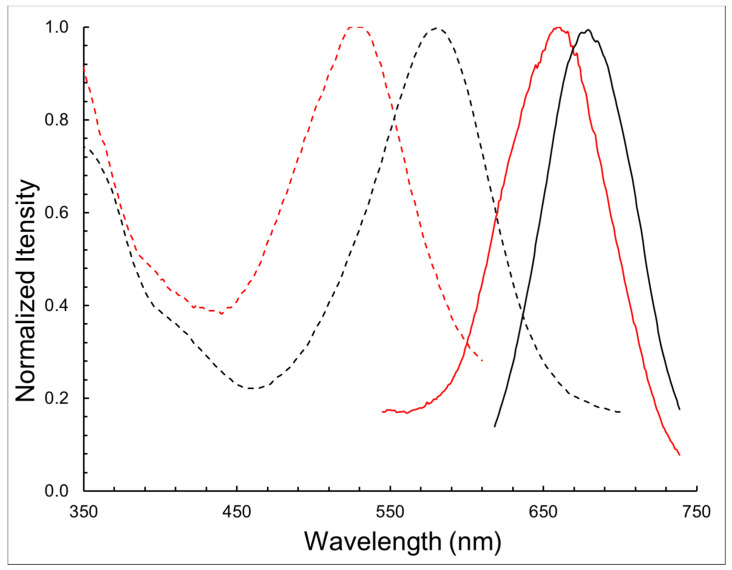
Normalized UV-vis absorption (dashed) and fluorescence emission (solid) spectra of BODIPY-COOH **1** (black) in MeOH and BODIPY dye **11** (red) in 1% CH_2_Cl_2_/MeOH.

The preparation of BODIPY dye **8** using ketopyrrole **7** and monoester **10** according to the procedure reported by Bochkov et al. [25] required POCl_3_ without any traces of HCl impurities and was difficult to reproduce because of instability of precursor **10** under acidic conditions. So, we tried to activate ketopyrrole derivative **7** by reaction with POCl_3_ during several hours in the absence of pyrrole **10**. After that, the reaction mixture was evaporated and a solution of monoester **10** in dichloromethane was added to the residue (Scheme 1). In this way, the condensation of derivative **7** with pyrrole carboxylic acid **10** was successful, though in some cases addition of 1–2 eq. of POCl_3_ and compound **10** was necessary. The yield of compound **8** was 89%. The compound was characterized by UV-vis, ^1^H- and ^13^C-NMR, and mass spectrometry. At the last stage (Scheme 1, step j), we carried out an acidic (aq. HCl/1,4-dioxane) hydrolysis of the methyl ester group of the BODIPY dye **8** linker arm and obtained the target BODIPY dye **1** in a high overall yield. The resulting compound was characterized by UV-vis, ^1^H and- ^13^C-NMR, and mass spectrometry. The UV-vis absorption spectra of **1** showed the bathochromic shift compared to the most BODIPY dyes [21,46,47]. Stokes shift is 98 nm, which is also higher than most common BODIPY dyes have (Table 1, Figure 1).

**Figure 2:**
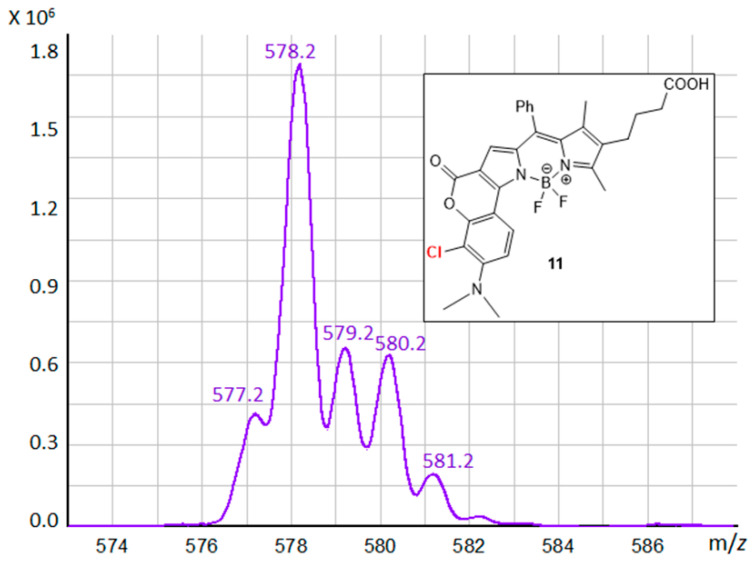
The suggested structure and isotope composition of [M + H]^+^ peak in ESI mass spectrum of the compound **11.**

Studying the acidic hydrolysis of BODIPY **8,** we found out that inadvertent using of distilled but not stabilized solvents such as tetrahydrofuran (THF) [48,49] in the course of aqueous HCl treatment of compound **8** resulted in some cases in a quantitative formation of a new “rose” fluorescent dye characterized by a *hypso*chromic shift in UV-vis electronic spectrum (Figure 1, Table 1), wherein Stocks shift was 134 nm, indicating a retention of the coumarin annulated core of the molecule. We assumed the influence of unidentified peroxide impurities present in non-stabilized THF during the acidic (aq. HCl) hydrolysis and proposed the structure for the new compound **11** (Figure 2) formed through oxidative chlorination of arenes [50,51,52]. ^1^H, ^13^C-NMR, and mass spectra of this compound were consistent with this hypothesis. Isotope composition in [M + H]^+^ signal in ESI-mass spectrum of the compound **11** indicated convincingly the presence of chlorine atom in the molecule (Figure 2).

The number and the structure of ^1^H-NMR signals of coumarin part of the molecule were changed indicating the disappearance of the H4 of the BODIPY aromatic system (see Supplementary Materials, compounds **1** vs. **11**). Similar changes in NMR spectra were detected for 8-Cl-coumarin [53] and 7,8 disubstituted coumarins [54,55].

In a separate experiment, we confirmed the possibility of oxidative chlorination of BODIPY dye **1** by peroxides under acidic (aq. HCl) conditions. BODIPY dye **1** was dissolved in a mixture of freshly distilled THF without peroxides and 6 N aq. HCl (5:1, *v*/*v*). *tert*-Butyl hydroperoxide (*t*-BuOOH) was added to the solution. In 3 h, the reaction was completed. The product isolated after work-up procedure and chromatographic purification was identical with the substance obtained earlier. If H_2_O_2_ was used instead of *t*-BuOOH, the same product was formed in 10 min accompanied by some decomposition. Increasing the reaction time or heating the reaction mixture led to a decomposition of the starting material and the products. In the absence of HCl, no reaction occurred except the slow degradation of BODIPY dye **1** to unidentified products. The same result was obtained while using another acid (1 mM aq. H_2_SO_4_) and *t*-BuOOH. 

A hypsochromic shift in absorption spectrum and an enhanced Stokes shift of BODIPY dye **11** in comparison with BODIPY **1** (Table 1) can hardly be explained by the chlorine substitution in coumarin ring [56]; however, similar effects were observed in a fused planar BODIPY [57]. Unusual spectral properties of BODIPY dye **11** make this compound interesting for further study.

**Scheme 3:**
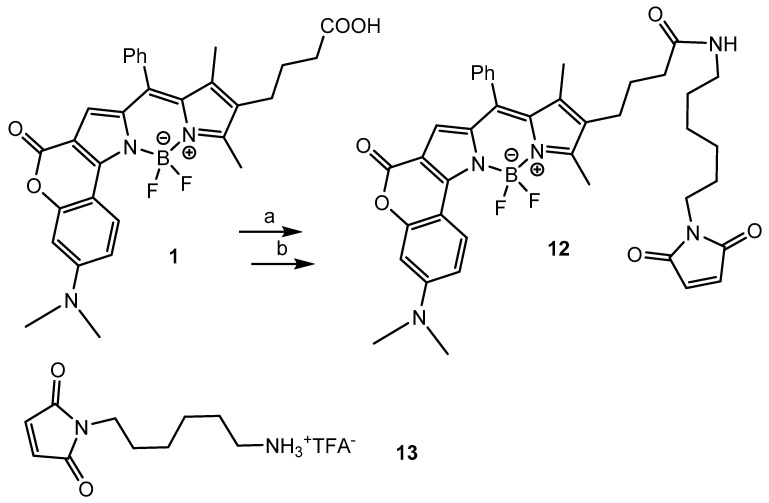
Synthesis of BODIPY-maleimide derivative. Reagents and conditions: a—TBTU, CH_2_Cl_2_, 10 min; b—maleimide **13**, TEA, 30 min.

Using a maleimide-cysteine “click” reaction is a widespread and effective method for conjugation of proteins with various reporter groups [58]. So, we prepared BODIPY maleimide derivative **12** for further conjugation with HSA. 1-Amino-6-*N*-maleimidohexane trifluoroacetate salt **13** was obtained according to the procedure reported by Horstmann et al. [59]. BODIPY-COOH dye **1** was activated by *N,N,N′,N′*-tetramethyl-*O*-(benzotriazol-1-yl)uronium tetrafluoroborate (TBTU) in CH_2_Cl_2_ and conjugated with maleimide **13** (Scheme 3, step b) in a presence of triethylamine (TEA). After work-up procedure the target compound **12** was purified by silica gel chromatography in a gradient of acetone in CH_2_Cl_2_ and precipitated with hexane. The yield of conjugate **12** was 50%. ^1^H- and ^13^C-NMR, and mass spectrometry data confirmed the structure of BODIPY-maleimide derivative **12**.

### 2.2. Bioconjugation

**Scheme 4:**
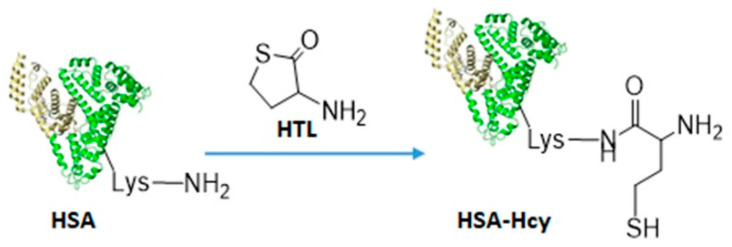
Thiol functionalization of the protein with homocysteine thiolactone (HTL).

In our work, we used the maleimide derivative BODIPY **12** for designing covalent HSA-based theranostic. The maleimide fragment mainly allows to attack cysteine residues in protein. HSA has 35 cysteine residues, with only Cys-34 is free for site-specific chemical modification [7]. But a titration of commercial HSA preparations with 5,5′-dithio-bis(2-nitrobenzoic acid (DTNB) indicated, that the sulfhydryl titer for most samples of HSA is approximately 30–40% [39,40]. The other 60–70% Cys-34 residues are manly reversibly oxidized as a mixed disulfide with cysteine or cysteinyl glycine, homocysteine and glutathione (in minor) [40]. In this work, we used the homocysteine thiolactone (HTL) as a covalent linker allowing us to obtain several additional free thiol groups in the HSA conjugate structure (Scheme 4).

HTL is a quite beneficial synthetic block for preparing different multifunctional agents [11,12,13,14,15,60,61]. HTL is susceptible to both nucleophilic and electrophilic attack because of its dual aminoacyl-thioester character. Self-condensation of two thiolactones (acylation of the free amino group of the one HTL molecule by the other HTL) can form the corresponding diketopiperazine derivative (3,6-bis(2-mercaptoethyl)piperazine-2,5-dione) [14,62,63,64]. Protecting of the amino group of thiolactone could maintain the integrity of the HTL ring. For this purpose, we used *N*-trifluoroacetyl homocysteine thiolactone. The trifluoroacetyl group offers an advantage of using the ^19^F MRI method to visualize the construct in vivo.

#### Synthesis of HSA-HcyTFAc-BODIPY Conjugate

**Scheme 5:**
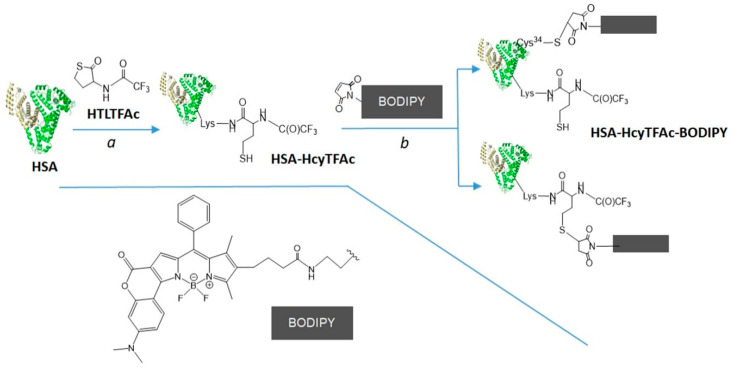
Synthetic path to HSA-HcyTFAc-BODIPY. Drug carrier (shown schematically as a heart-like bundle of helices)—HSA. A *N*-trifluoroacetylhomocysteine (HcyTFAc) is used as a functional handle. BODIPY dye shown schematically as a grey rectangle.

The synthesis of HSA-HcyTFAc-BODIPY conjugate was carried out in two steps (Scheme 5). The first step (a) involved homocysteinylation of the protein in PBS buffer (pH 7.4) at 37 °C. The method was adapted to that described in [14]. Low molecular weight homocysteine derivatives were removed from the HSA conjugate using a Millipore ultrafiltration tube (Amicon Centriprep YM30, Millipore, Bedford, MA, USA) having a molecular weight cut-off of 3000 Da. The resulting HSA-HcyTFAc was treated with maleimide derivative of BODIPY **12** in PBS buffer at 37 °C (Scheme 5, step b). Purification of the final product HSA-HcyTFAc-BODIPY was carried out using Millipore ultrafiltration tube, and then by ion exchange HPLC. The effectiveness of the two-step purification of the target covalent conjugate was confirmed by applying this procedure to a mixture of HSA and a BODIPY dye **1** that does not contain a maleimide fragment. According to data presented in Figure S1 in the Supplementary Materials, fraction 1 of ion exchange HPLC eluted with 0% of ethanol corresponds to the HSA protein free from BODIPY dye. This is proved by the absence of an absorption band at 600 nm in the UV-vis spectrum of this fraction. 

**Figure 3:**
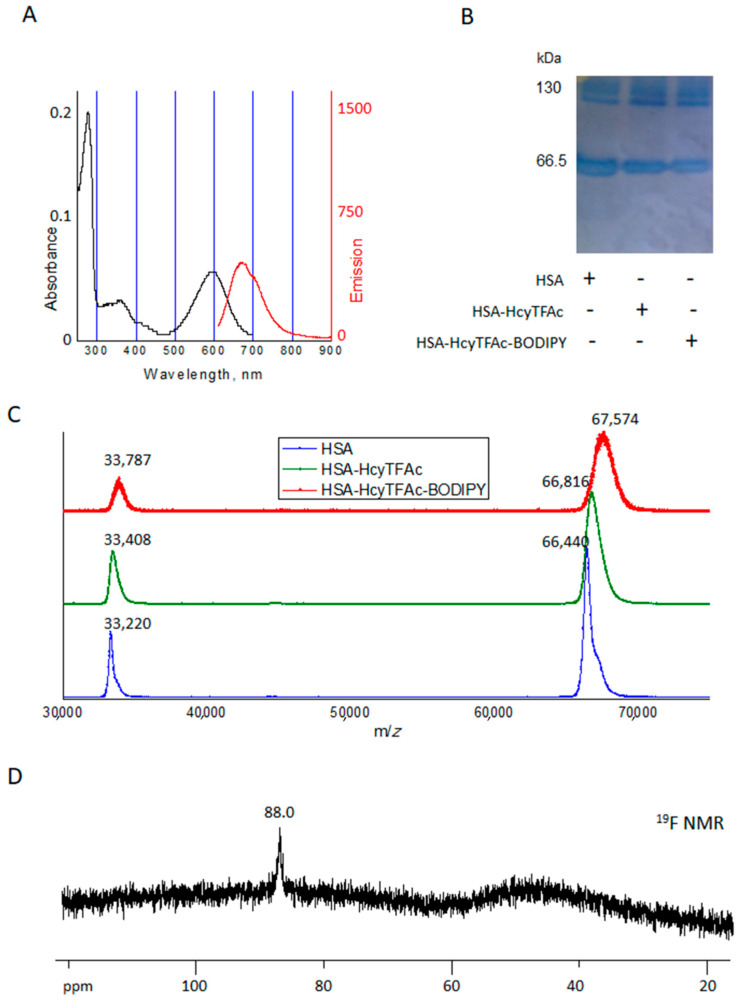
(**A**) UV-vis spectra (black line), and fluorescent spectra (red line) of HSA-HcyTFAc-BODIPY, PBS buffer, pH 7.4, excitation wavelength 590 nm. (**B**) SDS-PAGE of homocysteinamide conjugates of the HSA under Laemmle condition with subsequent Coomassie blue staining. (**C**) MALDI-TOF spectra of HSA (blue line), HSA-HcyTFAc (green line) and HSA-HcyTFAc-BODIPY (red line). (**D**) 19F NMR spectrum (at 282.4 MHz) of HSA-HcyTFAc-BODIPY (0.6 mM) in PBS buffer at 37 °C, pH 7.4; to provide deuterium lock, D2O was added to 20% of the total volume. The chemical shifts are referred to the resonance of C6F6 at 0.0 ppm.

Characteristics of the resulting HSA-HcyTFAc-BODIPY are shown in Figure 3. 

Changes in the molecular masses of the HSA and its conjugates were monitored with MALDI-TOF mass spectrometry. HSA in a native form is a monomer of 585 amino acid residues [65]. Plasma-derived HSA generally exhibits a broad range of post-translationally modified forms (glycated, truncated, oxidated, or genetic variants) [39,40,66,67]. The measurements for the [M + H]^+^ ions *m*/*z* values with MALDI-TOF mass analyzers used in these experiments do not have great accuracy for masses over 60 kDa Additionally, the different post-translationally modified forms can cause spectral overlap. MALDI mass spectra for each sample were recorded in four replicates. The molecular mass of the HSA A3782 (Sigma-Aldrich, St. Louis, MO, USA) in our mass experiments averaged 66.440 and 33.220 kDa for the double charged protein (Figure 3C, blue line).

The HSA-HcyTFAc conjugate had a measured molecular mass of 66.816 and 33.408 kDa for the double charged protein (Figure 3C, green line). The difference between the homocysteinylated and native species was 376 Da, which corresponds to 1.8 *N*-trifluoroacetylhomocysteinyl moieties, *N*-linked by amide linkages to Lys residues of HSA (*N*-Lys-HcyTFAc; 214 Da). The presence of trifluoroacetyl groups was confirmed by ^19^F NMR data (Figure 3D). The ^19^F NMR spectrum shows a signal at 88.0 ppm, corresponding to fluorine atoms included in the *N*-trifluoroacetylhomocysteine residues.

The HSA-HcyTFAc-BODIPY conjugate had a measured molecular mass of 67.574 and 33.787 kDa for the double charged protein (Figure 3C, red line). The difference between the HSA-HcyTFAc-BODIPY and HSA-HcyTFAc (Figure 3C, green line) was 758 Da, which corresponds to 1 BODIPY dye *S*-linked to SH residue of HSA: Cys34, or free SH group of HcyTFAc residues (linked maleimide BODIPY derivative **12**; 721 Da). The BODIPY residue can be connected to the *N*-trifluoroacetylhomocysteinylated HSA via Cys-34 as far as *N*-trifluoroacetylhomocysteine thiol group (Scheme 5).

All HSA samples were assessed by SDS-PAGE and Coomassie blue staining. All of them showed major bands at approximately 65 kDa, similar to the published molecular weight for HSA of 66.5 kDa [65] (Figure 3B). Our starting protein, HSA A3782, was ~76% monomeric with MW ~66.5 kDa (Table 2). *N*-Trifluorohomocysteinylation of lysine residues produced new thiol groups in a protein. This makes *N*-HcyTFAc-HSA more susceptible to oxidation than HSA, and the amount of aggregates increases [68]. The total amount of oligomers increased to ~57% in the conjugates from ~23% in the starting HSA (Figure 3B, Table 2). Further addition of the BODIPY dye to the conjugate increased the amount of oligomers to ~67%. The aggregates formed were probably obtained due to the formation of S-S bonds between monomeric forms of the HSA in the course of homocysteinelation, since the number of higher aggregates decreased while performing SDS PAGE in the presence of dithiotreitol (DTT) (Table 2).

UV-vis spectra of the HSA-HcyTFAc-BODIPY (Figure 3A, black line) revealed the appearance of absorption bands corresponding to a BODIPY dye in the structure of the HSA-HcyTFAc-BODIPY conjugate (357 and 595 nm) along with the absorption band corresponding to HSA (278 nm). The conjugation of the BODIPY dye to HSA gives a protein fluorescent conjugate with large Stokes shift (80 nm). Excitation at 590 nm gives fluorescence maximum at 670 nm (Figure 3A, red line). Thus, the attachment of the BODIPY to the protein core does not lead to significant deterioration of the dye fluorescent properties (Table 1).

### 2.3. In Vitro Cytotoxicity Assay

**Figure 4:**
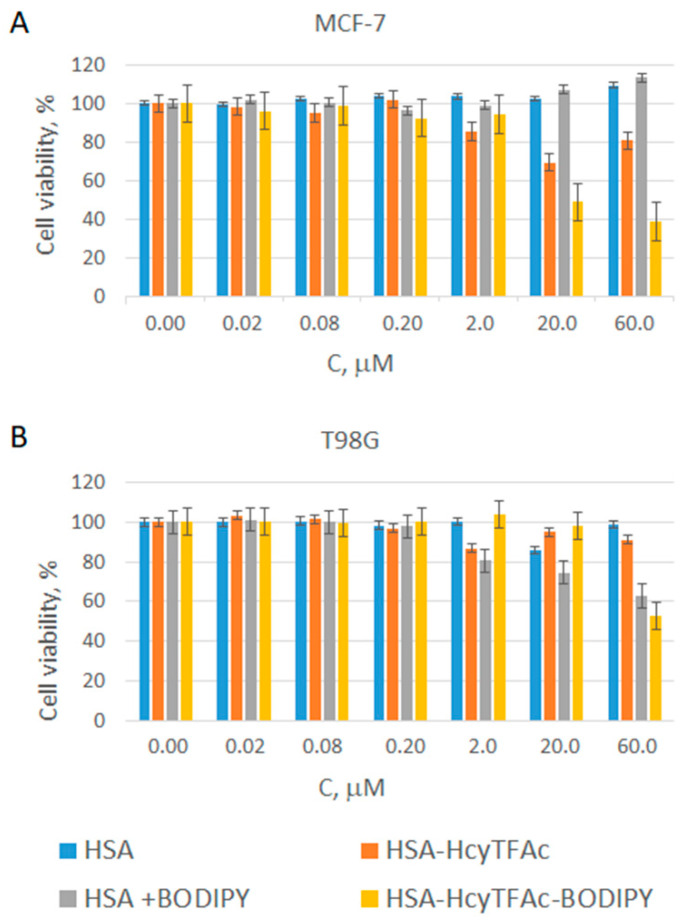
Cytotoxicity of the HSA and its conjugates. (**A**) breast cancer cell line MCF 7; (**B**) glioma cell line T98G.

The cytotoxicity of HSA conjugates was evaluated in vitro by MTT assay [69]. The results of the toxicity studies are shown in Figure 4. It was shown that the toxicity of the conjugate HSA-HcyTFAc-BODIPY was slightly higher on breast cancer cells (MCF-7, Figure 3A, 40%) than on glioma cells (T98G, Figure 3B). Glioma cells showed moderate viability (60%) under the influence of the conjugate HSA-HcyTFAc-BODIPY. Anyway, the toxicity of the covalent conjugate was somewhat greater than that of the HSA + BODIPY mixture, or HSA-HcyTFAc. We expect that neutron irradiation will significantly decrease the viability of both cell lines. This work is now underway.

## 3. Materials and Methods 

We used boron trifluoride diethyl etherate, 10% palladium on activated charcoal, bromine, sodium azide (Sigma-Aldrich, St. Louis, MO, USA); zinc (Panreac, Barcelona, Spain); *O*-(benzotriazol-1-yl)-*N,N,N′,N′*-tetramethyluronium tetrafluoroborate (Merck, Darmstadt, Germany). Diphenyl malonate [25], (benzoylmethylene)triphenylphosphorane [70], ethyl 4-(4-ethoxy-4-oxobutyl)-3,5-dimethyl-1H-pyrrole-2-carboxylate **9** [43], 4-methoxycarbonylpropyl-3,5-dimethylpyrrole-2-carboxylic acid **10** [45], and 1-amino-6-*N*-maleimidohexane trifluoroacetate salt **13** [59] were synthesized as previously reported, and spectroscopic characteristics were in full agreement with the reported data. For the synthesis of compounds **3**–**7**, see Supplementary Materials. All solvents were purchased from Reachem (Moscow, Russia). Organic solvents were dried and purified by standard procedures if necessary.

All buffer solutions were prepared with doubly deionized water. Bioconjugation experiments were carried out in standard polypropylene Eppendorf^®^ safe-lock tubes (1.5 mL) at atmospheric pressure with mixing at 37 °C. Conjugation buffer was phosphate-buffered saline (PBS) (12 mM phosphates, 140 mM NaCl at pH 7.4). Low molecular weight materials were removed from the protein conjugate solution by ultrafiltration at 10,000 rpm twenty-four times using a Millipore ultrafiltration tube with 3000 Da cut off Amicon Centriprep YM30 (Millipore, Bedford, MA, USA). HSA was purchased from Sigma-Aldrich Chem. Co. (St. Louis, MO, USA). The concentration of protein solutions was determined at 294 nm, pH 13, using the molar extinction coefficient ε = 4.44 × 10^4^ M^−1^cm^−1^ with a UV-1800 spectrometer (Shimadzu, Kyoto, Japan). 

All HSA conjugates were analyzed by sodium dodecyl sulfate polyacrylamide gel electrophoresis (7% PAAG, under Laemmli condition) with subsequent Coomassie Brilliant Blue (Bio-Rad, Hercules, CA, USA) staining. Quantitative data were obtained by digitizing the gel using GelPro Analyzer software (Media Cybernetics, Rockville, MD, USA). Ion exchange HPLC was carried out using NGC Chromatography System (BIO RAD, Hercules, CA, USA). UV detection at 280 nm. Polysil CA-500 column, 10 μm, 250 × 4.6 mm. Elution: 0.3 M phosphate buffer pH 7.4, a linear gradient of ethanol in water 0→70%.

NMR spectra were acquired on Bruker AV-400, AV-300, and AV-500 instruments (Bruker Daltonics, Billerica, MA, USA) in appropriate deuterated solvents at 30 and 37 °C (in the case of the protein conjugate). Chemical shifts (δ) are reported in ppm relative to the tetramethylsilane (TMS) or C_6_F_6_ signals. Coupling constants *J* are reported in Hertz. 

MALDI-TOF was registered on Autoflex III mass spectrometer (Bruker Daltonics, Billerica, MA, USA) and ESI mass spectra was registered on Agilent ESI MSD XCT Ion Trap (Agilent Technologies, Santa Clara, CA, USA) at The Joint Center for genomic, proteomic, and metabolomics studies (ICBFM SB RAS, Russia). All mass spectra were registered in positive mode. A smart beam-II laser was used for MALDI-TOF mass. Protein samples were desalted with ZipTip C4 pipette tips. The protein sample solution (2 μL, 6 × 10^−4^ M) was mixed with 2 μL of aqueous 2% trifluoroacetic acid (TFA). The matrix (2,5-dihydroxyacetophenone, 2 μL) was added to the latter solution. The mixture was pipetted up and down until the crystallization started. Mass spectra were obtained by averaging 3000 laser shots. External calibration was provided by [M + H]^+^ HSA at *m*/*z* 66.5 kDa.

Fluorescent emission and absorption spectra of the compounds **1** and **11** were recorded in 1% CH_2_Cl_2_/MeOH and MeOH at 25 °C with CLARIOStar Plus (BMG Labtech, Offenburg, Germany). Fluorescence emission spectrum of the protein conjugate HSA-HcyTFAc-BODIPY was recorded in PBS at 25 °C with Cary Eclipse fluorimeter (Agilent Technologies, Santa Clara, CA, USA) in the range of 600–900 nm using the excitation wavelength *λ*_abs_ = 590 nm.

Monitoring of a reaction progress (except bioconjugation) was performed on a Milichrom A02 chromatograph system equipped with the MultiChrom program package (Econova, Novosibirsk, Russia) on a ProntoSIL 125 C18 column (2 × 75 mm) in a gradient of buffer B (0.1 M triethylammonium acetate (TEAAc), pH 7.0, 80% MeCN) in buffer A (0.1 M TEAAc, pH 7.0, water) with an elution rate of 0.2 mL/min and UV detection at 250, 260, 280, and 300 nm. TLC was carried out on Kieselgel 60 F_254_ plates (Merck, Darmstadt, Germany) in the proper solvent systems and visualized by UV irradiation or ninhydrin test. Preparative silica gel column chromatography was performed using silica gel 60 (40–63 μm/230–400 mesh) (Macherey-Nagel, Düren, Germany). Polysil SA-500 10 μm for ion-exchange chromatography column was purchased from NanoTech-S LLS (Novosibirsk, Russia).

### 3.1. BODIPY Compound ***8***

Phosphoryl oxychloride (0.45 mL, 4.8 mmol, 5 eq.) was added to a stirred suspension of ketopyrrole **7** (0.100 g, 0.3 mmol) in CH_2_Cl_2_ (4 mL). The reaction mixture was stirred at RT for 48 h. The solvent and POCl_3_ excess were evaporated, the residue was co-evaporated with toluene and dried in vacuum. The residue was suspended in CH_2_Cl_2_ (4 mL) and solution of pyrrole **10** (0.100 g, 0.4 mmol) in CH_2_Cl_2_ was added. The reaction mixture was stirred at RT for 48 h. Thereafter, second portion of pyrrole **10** (0.100 g, 0.4 mmol) was added and the reaction mixture was stirred for an additional 2 h. Then, triethylamine (0.60 mL, 4.3 mmol) was added and stirring continued for 15 min, then BF_3_·Et_2_O (1.1 mL, 9.0 mmol) was added and reaction mixture was further stirred at RT for 48 h. The reaction mixture was quenched with water. Organic layer was separated, evaporated at reduced pressure and the residue was purified on a silica gel using gradient of THF in toluene (0–10%) as an eluent. The appropriate fractions were evaporated. Yield: 0.149 g, 0.27 mmol, 89%. ^1^H-NMR (CDCl_3_): 8.56 (1H, d, *J* 9.1, H1), 7.52–7.45 (3H, m, *m,p*-Ph), 7.34–7.30 (2H, m, *o*–Ph), 6.92 (1H, s, H7), 6.73 (1H, dd, *J* 9.3, 2.5, H2), 6.55 (1H, d, *J* 2.3, H4), 3.65 (3H, s, OCH_3_), 3.06 (6H, s, N(CH_3_)_2_), 2.72 (3H, s, CH_3_), 2.41 (2H, t, *J* 7.7, CH_2_), 2.33 (2H, t, *J* 7.0, CH_2_), 1.78–1.68 (2H, m, CH_2_), 1.48 (3H, s, CH_3_); ^13^C-NMR (CDCl_3_): 173.20, 164.08, 159.17, 155.66, 152.22, 148.45, 142.62, 140.37, 137.02, 134.85, 134.49, 133.37, 129.40, 128.70, 128.49, 125.76, 113.14, 109.19, 102.46, 98.79, 51.48, 39.90, 33.04, 24.45, 23.12, 14.00, 13.50. MS ESI (*m*/*z*): [M + H]^+^ calcd. for C_31_H_31_BF_2_N_3_O_4_ 558.24; found 557.80.

### 3.2. BODIPY-COOH ***1***

HCl (aq., conc., 4 mL) was added to a solution of BODIPY **8** (0.02 g, 0.04 mmol) in 1,4-dioxane (20 mL). The reaction mixture was stirred at RT for 6 h, then diluted with CH_2_Cl_2_ (12 mL). The reaction mixture was neutralized with 1 M TEAB (80 mL). Organic layer was separated, washed with water (80 mL), and evaporated. The residue was dried in vacuum. Yield: 0.02 g, 0.037 mmol, 92%. ^1^H-NMR (DMSO-*d*_6_): 8.41 (1H, d, *J* 9.2, H1), 7.66–7.56 (3H, m, *m,p*-Ph), 7.54–7.49 (2H, m, *o*–Ph), 6.87 (1H, dd, *J* 9.3, 2.5, H2), 6.63 (1H, d, *J* 2.5, H4), 6.60 (1H, s, H7), 3.06 (6H, s, N(CH_3_)_2_), 2.70 (3H, s, CH_3_), 2.44 (2H, t, *J* 7.8, CH_2_), 2.25 (2H, t, *J* 6.8, CH_2_), 1.67–1.58 (2H, m, CH_2_), 1.49 (3H, s, CH_3_); ^13^C-NMR (DMSO-*d*_6_): 174.04, 165.76, 157.59, 155.19, 152.36, 147.06, 143.03, 139.55, 136.39, 136.23, 134.31, 132.81, 129.76, 128.91, 128.70, 126.74, 123.50, 112.13, 109.39, 101.18, 98.38, 39.63, 32.89, 24.20, 22.53, 13.51, 12.23; MS ESI (*m*/*z*): [M + H]^+^ calcd. for C_30_H_29_BF_2_N_3_O_4_ 544.22; found 543.80. 

### 3.3. BODIPY Compound ***11***

BODIPY-COOH **1** (0.02 g, 0.037 mmol) was dissolved in a mixture of freshly distilled THF and 6 N aq. HCl (5:1, total volume 6 mL). *t*-BuOOH (0.5 mL, 5.56 mmol) was added to the solution. In 3 h, the reaction mixture was neutralized with 1 M TEAB (4 mL) and diluted with CH_2_Cl_2_ (20 mL). Organic layer was separated, washed with water (10 mL), and evaporated. The product was purified on a silica gel using gradient of EtOH in CH_2_Cl_2_ (0–10%) as an eluent. Appropriate fractions were combined and evaporated. The residue was dissolved in CH_2_Cl_2_ and precipitated with hexane. Yield: 0.02 g, 0.035 mmol, 94%. ^1^H-NMR (CDCl_3_): 8.58 (1H, d, *J* 9.1, H1), 7.54–7.48 (3H, m, *m,p*-Ph), 7.36–7.32 (2H, m, *o*-Ph), 7.04 (1H, d, *J* 9.1, H2), 6.92 (1H, s, H7), 2.93 (6H, s, N(CH_3_)_2_), 2.73 (3H, s, CH_3_), 2.39–2.32 (4H, m, CH_2_), 1.76–1.68 (2H, m, CH_2_), 1.48 (3H, s, CH_3_); ^13^C-NMR (CDCl_3_): 177.30, 167.14, 158.01, 152.83, 150.62, 146.70, 144.13, 140.89, 137.61, 135.75, 135.35, 132.95, 129.72, 128.71, 128.62, 124.40, 124.29, 114.97, 113.99, 113.34, 108.61, 43.32, 32.72, 23.44, 23.06, 13.80, 12.60; MS ESI (*m*/*z*): [M + H]^+^ calcd. for C_30_H_28_BClF_2_N_3_O_4_ 578.18; found 578.20. 

### 3.4. BODIPY-Maleimide ***12***

BODIPY-COOH **1** (0.025 g, 0.005 mmol) and TBTU (0.032 g, 0.01 mmol) were dissolved in DMF (0.5 mL). In 10 min, TEA (0.045 mL, 0.32 mmol) and 1 M solution of amine trifluoroacetate salt **12** in CH_2_Cl_2_ (0.1 mL) were added to the reaction mixture. In 2 h, the reaction mixture was diluted with CH_2_Cl_2_ (10 mL) and washed with 5% aq. NaHCO_3_. Organic layer was separated, evaporated at reduced pressure, and the residue was purified on a silica gel using gradient of acetone in CH_2_Cl_2_ (0–20%) as an eluent. The appropriate fractions were evaporated. Yield: 0.01 mg, 0.014 mmol, 30%. ^1^H-NMR (CDCl_3_): 8.56 (1H, d, *J* 9.2, H1), 7.51–7.45 (3H, m, *m,p*-Ph), 7.33–7.29 (2H, m, *o*-Ph), 6.91 (1H, s, H7), 6.73 (1H, dd, *J* 9.2, 2.7, H2), 6.66 (2H, s, -CH=), 6.55 (1H, d, *J* 2.7, H4), 5.48 (1H, t, *J* 5.7, NH), 3.48 (2H, t, -CH_2_N<), 3.20 (2H, app. q, -CH_2_NH), 3.06 (6H, s, N(CH_3_)_2_), 2.72 (3H, s, CH_3_), 2.43–2.38 (2H, m, CH_2_), 2.17 (2H, t, *J* 7.3, CH_2_), 1.77–1.71 (2H, m, CH_2_), 1.59–1.53 (2H, m, CH_2_), 1.48, (3H, s, CH_3_), 1.35–1.25 (6H, m, CH_2_); ^19^F NMR (CDCl_3_): 18.96 (q, *J* 32.8); ^13^C-NMR (CDCl_3_): 171.81, 170.83, 164.42, 159.33, 155.70, 152.24, 148.43, 142.69, 140.30, 137.07, 135.30, 134.62, 133.95, 133.42, 129.47, 128.75, 128.55, 127.30, 125.68, 113.09, 109.26, 102.50, 98.84, 40.03, 39.12, 37,37, 35.47, 29.59, 29.26, 28.23, 25.94, 25.24, 23.32, 13.68, 12.57; MS ESI (*m*/*z*): [M+H]^+^ calcd. for C_40_H_43_BF_2_N_5_O_5_ 722.33; found 722.40. 

### 3.5. HSA-HcyTFAc

The synthesis of mHSA-HsyTFAc was adapted from [14]. The conjugation reaction was carried out in the mixture of PBS buffer (pH 7.4, 1.7 mM KH_2_PO_4_, 5.2 mM Na_2_HPO_4_, 150 mM NaCl) and DMSO (20: 1 *v*/*v*) at 37 °C, during 42 h in the dark. HSA diluted in PBS (0.84 mM, 1 mL, 0.84 µmol) was mixed with 6.5-fold excess of HTLTFAc diluted in DMSO (0.109 M, 0.05 mL, 5.46 µmol). When the reaction time is over low molecular weight homocysteine derivatives were removed from the HSA conjugates using a Millipore ultrafiltration tube (Amicon Centriprep YM30, Millipore, Bedford, MA) having a molecular weight cut-off of 3000 Da. The yield of HSA- HcyTFAc derivatives was ~55%. UV-vis (NaOH, pH 12): λ_max_ 296 nm (ε = (4.4 ± 0.1) × 10^4^). The molecular weight of the conjugate was determined by MALDI-TOF analysis, and differences in MW were used to calculate the number of HcyTFAc bound per albumin. MS (MALDI ToF) *m*/*z*: Calculated for HSA-HcyTFAc (MW HSA + MW HcyTFAc), 66.654 kDa, found 66.816 kDa. Note: the calculated value was obtained as 66.440 kDa (measured MW of reference HSA) + 214 Da (calculated MW of HcyTFAc); 162 Da the error between calculated and found MS (MW for 0.76 residues of HcyTFAc)

### 3.6. HSA-HcyTFAc-BODIPY

A solution (0.5 mL, 0.7 mM, 0.35 μmol) of the HSA-HcyTFAc in PBS buffer (pH 7.4) was mixed with BODIPY-maleimide **12** in DMSO (25 μL, 0.084 M, 2.1 μmol,). The reaction mixture was incubated under constant gently stirring at 37 °C in the dark for 17 h. The protein conjugates were purified using a Millipore ultrafiltration tube (Amicon Centriprep YM30, Millipore, Bedford, MA) having a molecular weight cut-off of 3000 Da, further using ion exchange HPLC, and stored at 4 °C. The yield of HSA-HcyTFAc-BODIPY derivatives was ~90%. UV-vis (PBS buffer, pH 7.4): λ_max_ 278 nm (ε = (4.31 ± 0.1) × 10^4^), λ_max_ 357 nm (ε = (1.51 ± 0.1) × 10^4^), λ_max_ 595 nm (ε = (2.21 ± 0.1) × 10^4^). ^19^F NMR (PBS buffer, pH 7.4, D_2_O to 20% of the total volume) 88.0 (*N*-trifluoroacetyl residue). The molecular weight of the conjugate was determined by MALDI-TOF analysis, and differences in MW were used to calculate the number of BODIPY bound per albumin. MS (MALDI TOF) *m*/*z*: Calculated for HSA-HcyTFAc-BODIPY (MW HSA + 1.76 MW HcyTFAc + MW BODIPY), 67.537 kDa, found 67.574 kDa. Note: the calculated value was obtained as 66.440 (measured MW of reference HSA) + 364 (calculated 1.76 MW of HcyTFAc) + 721 (calculated MW of BODIPY).

### 3.7. In Vitro Cytotoxicity Assay

The cytotoxicity of HSA conjugates was evaluated in vitro by MTT test [69]. Exponentially growing cells were plated in 96-well plates (~2000 cells per well) and were incubated for 24 h at 37 °C in RPMI-1640 (5% CO_2_). The solutions of HSA and its conjugates were applied in medium with protein equivalent concentrations ranging from 0.02–60 μM. Time of the cell viability evaluation was 72 h after the start of the treatments. An aliquot (10 μL) of MTT solution (25 mg/mL in PBS) was added to each well, and the plates were incubated at 37 °C for 3 h. The medium was removed, and the crystals of formazan that had formed were dissolved in 100 μL of isopropanol. The absorbance at 570 nm (peak) and 620 nm (baseline) was read using a microplate reader Multiscan FC (Thermo Fisher Scientific, Waltham, MA, USA). Results were expressed as a percentage to the control values. All values in Figure 3 of the present study are given as mean ± standard deviation (S.D.) values, and all measurements were repeated three times.

## 4. Conclusions

This study was directed toward the development of the HSA-based multimodal platform for the creation of a new BODIPY containing HSA-based theranostic with improved spectral properties for BNCT.

Boron containing BODIPY dye with large Stokes shift (98 nm) and maleimide arm was designed and synthesized. Thiol-‘click’ chemistry was used to prepare conjugate of the BODIPY dye with human serum albumin (HSA). Covalent connection of this BODIPY residue to *N*-trifluoroacetylhomocysteinylated HSA gives the fluorescent boron-containing protein conjugate (HSA-HcyTFAc-BODIPY) with Stokes shift of about 80 nm. Conjugation of boron compounds and HSA (carrier protein with a long plasma half-life) is expected to extend a time circulation of boron compounds in the body and to ensure the accumulation of the boron in the tumor. 

Our design strategy allows combining in future within one HSA-based theranostic a therapeutic group suitable for BNCT, a fluorescent dye with a large Stokes shift with characteristics suitable for FLECT CT, and fluorine atoms, which is promising for MRI. All this makes it possible to create, in the future, the theranostic allowing determination of the location of the conjugates in vivo, in real time by FLECT/CT images, reducing the number of animals required for the fast investigation of new nanosystems as chemotherapeutic agent and BNCT drug candidates. 

## Figures and Tables

**Table 1:** UV-vis absorption and fluorescence data for new BODIPY dyes.

Compound	λ_abs_ (nm)	λ_em_ (nm)	log ε	∆λ (nm)
BODIPY **1**	580	678	4.54	98
BODIPY **11**	528	662	4.32	134

**Table 2:** Quantitative data of the SDS–PAGE analysis of HSA conjugates ^a^.

Conditions	HSA Type	Higher Aggregates	Monomer~66.5 kDa
	HSA	23.5	76.5
Without DTT	HSA-HcyTFAc	57.2	42.8
	HSA-HcyTFAc-BODIPY	66.7	33.3
With DTT (data not shown)	HSA	16.5	83.5
	HSA-HcyTFAc	15.4	84.6
	HSA-HcyTFAc-BODIPY	14.8	85.2

^a^ Values are given as percentage of intensity to total intensity in the lane (%). Quantitative data were obtained by digitizing the gel using GelPro Analyzer software (Media Cybernetics).

## Supplementary Materials

The following are available online. NMR and mass spectra of all synthesized compounds. Ion-exchange chromatography of the mixture HSA + BODIPY 1.

## Sample Availability

Samples of the compounds **1**, **11**, **12** are available from the authors.
