# Supplementary material for: Human Serum Albumin Labelling with a New BODIPY Dye Having a Large Stokes Shift
Source: Molecules. 2021 May 3;26(9):2679. doi: 10.3390/molecules26092679 (PMC8124464; doi:10.3390/molecules26092679)
Supplement: Supplementary file 1 [file molecules-26-02679-s001.zip › molecules-1208158-supplementary.pdf]

Human Serum Albumin Labelling with a New BODIPY Dye Having a Large Stokes Shift

Valeria I. Raskolupova [1,2], Tatyana V. Popova [1,2], Olga D. Zakharova [1], Anastasia E. Nikotina[1,2], Tatyana V. Abramova [1,*] and Vladimir N. Silnikov [1]

[1] Institute of Chemical Biology and Fundamental Medicine SB RAS, Lavrent'ev Ave, 8, 630090 Novosibirsk, Russia; v.raskolupova@mail.ru (V.I.R.); popovatv@niboch.nsc.ru (T.V.P.); isar@niboch.nsc.ru (O.D.Z.); a.nikotina@mail.ru (A.E.N.); abramova@niboch.nsc.ru (T.V.A.); silnik@niboch.nsc.ru (V.N.S.)

[2] Novosibirsk State University, Pirogova St., 2, 630090 Novosibirsk, Russia; v.raskolupova@mail.ru (V.I.R.); popovatv@niboch.nsc.ru (T.V.P.); a.nikotina@mail.ru (A.E.N.)

* Correspondence: abramova@niboch.nsc.ru; Tel.: +7-383-363-51-83 (T.V.A.)

Synthetic protocols and spectral data for new compounds

7-(Dimethylamino)-4-hydroxy-2H-chromen-2-one (3)

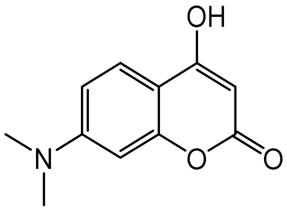

Solution of diphenyl malonate (2.36 g, 10 mmol) and 3-(*N,N*-dimethylamino)phenol (1.52 g, 16 mmol) in toluene (30 mL) was refluxed for 10 h. The reaction mixture was cooled and crystals formed were filtered off. The crude product was recrystallized from ethanol. Yield: 1.84 g, 9.0 mmol, 89%. ^{1}H NMR ($CDCl_3$): 7.56 (1H, d, *J* 8.9, H5), 6.68 (1H, dd, *J* 9.0, 2.2, H6), 6.48 (1H, d, *J* 2.1, H8), 5.28 (1H, s, H3), 2.99 (6H, s, $N(CH_3)_2$).

^{1}H NMR

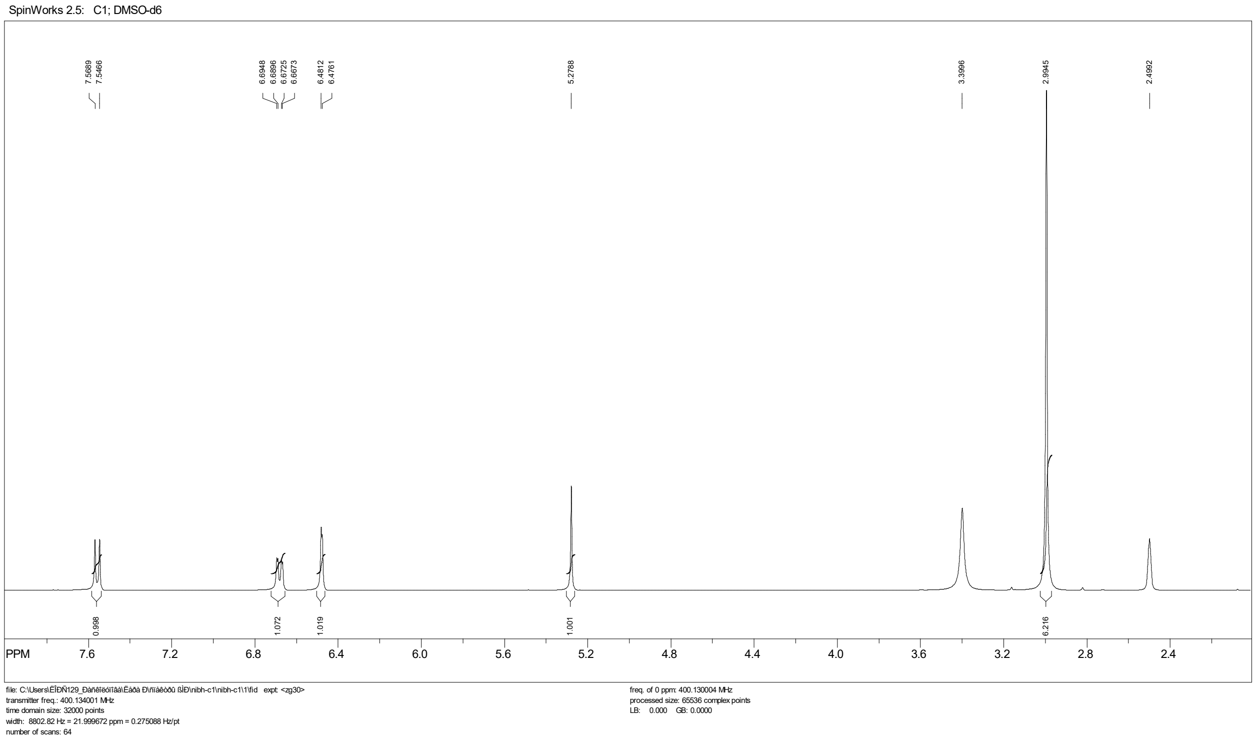

4-Chloro-7-(dimethylamino)-2-oxo-2H-chromene-3-carbaldehyde (4)

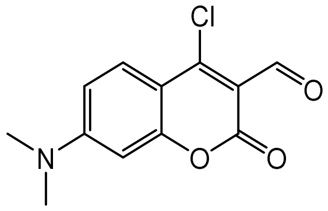

Phosphorus oxychloride (1.60 mL, 17 mmol) was added to a solution of hydroxycoumarin (**3**) (1.31 g, 6.4 mmol) in DMF (5 mL) and stirred at RT for 24 h. Then the reaction mixture was cooled, poured into 0.6 M aqueous sodium acetate and stirred for 1 h. The precipitate formed was filtered off and washed with water to give crude aldehyde which was recrystallized from ethanol. Yield: 1.32 g, 5.2 mmol, 82%. Orange crystals. ^{1}H NMR (DMSO-*d6*): 9.70 (1H, s, CHO), 7.70 (1H, d, *J* 9.2, H5), 6.79 (1H, dd, *J* 9.2, 2.4, H6), 6.51 (1H, d, *J* 2.3, H8), 3.10 (6H, s, $N(CH_3)_2$).

^{1}H NMR

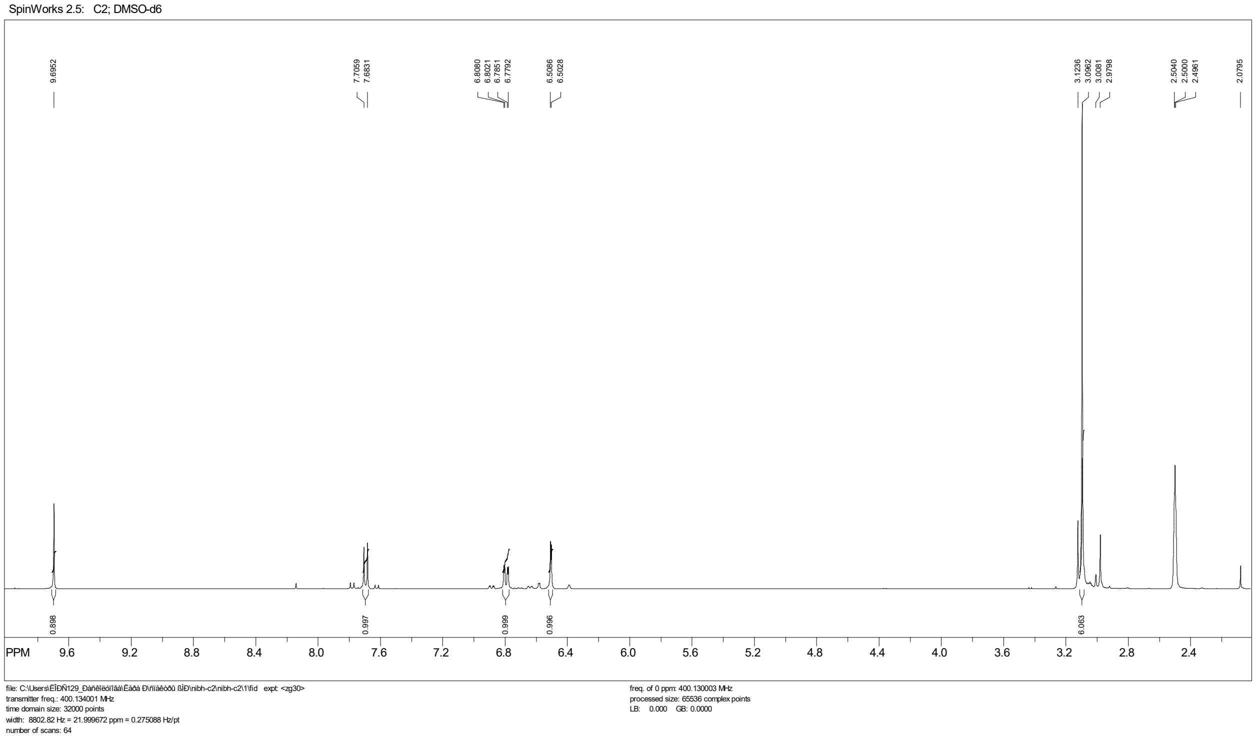

(E)-4-Chloro-7-(dimethylamino)-3-(3-oxo-3-phenylprop-1-en-1-yl)-2H-chromen-2-one (5)

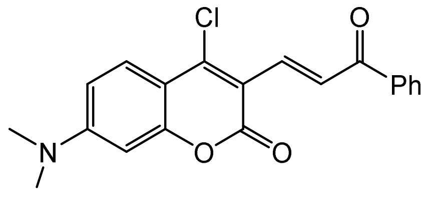

Coumarin (**4**) (1.32 g, 5.3 mmol) and (benzoylmethylene)triphenylphosphorane (2.02 g, 5.3 mmol,) were dissolved in CH_2Cl_2 (30 mL) and stirred at RT for 24 h. The solvent was removed under reduced pressure and the residue was purified on a silica gel using gradient of EtOH in CH_2Cl_2 (0-10%) as an eluent. The appropriate fractions were evaporated and then recrystallized from ethanol/acetonitrile (5/2) mixture to give orange crystals. Yield: 0.650 g, 1.8 mmol, 35%. ^{1}H NMR ($CDCl_3$): 8.37 (1H, d, *J* 15.2, CH=CH), 8.05 (1H, d, *J* 15.2, CH=CH), 8.01-7.97 (2H, m, *o*-Ph), 7.66 (1H, d, *J* 9.2, H5), 7.49 (1H, t, *J* 7.3, *p*-Ph), 7.41 (2H, t, *J* 7.4, *m*-Ph), 6.60 (1H, dd, *J* 9.1, 2.4, H6), 6.38 (1H, d, *J* 2.4, H8), 3.03 (6H, s, $N(CH_3)_2$).

^{1}H NMR

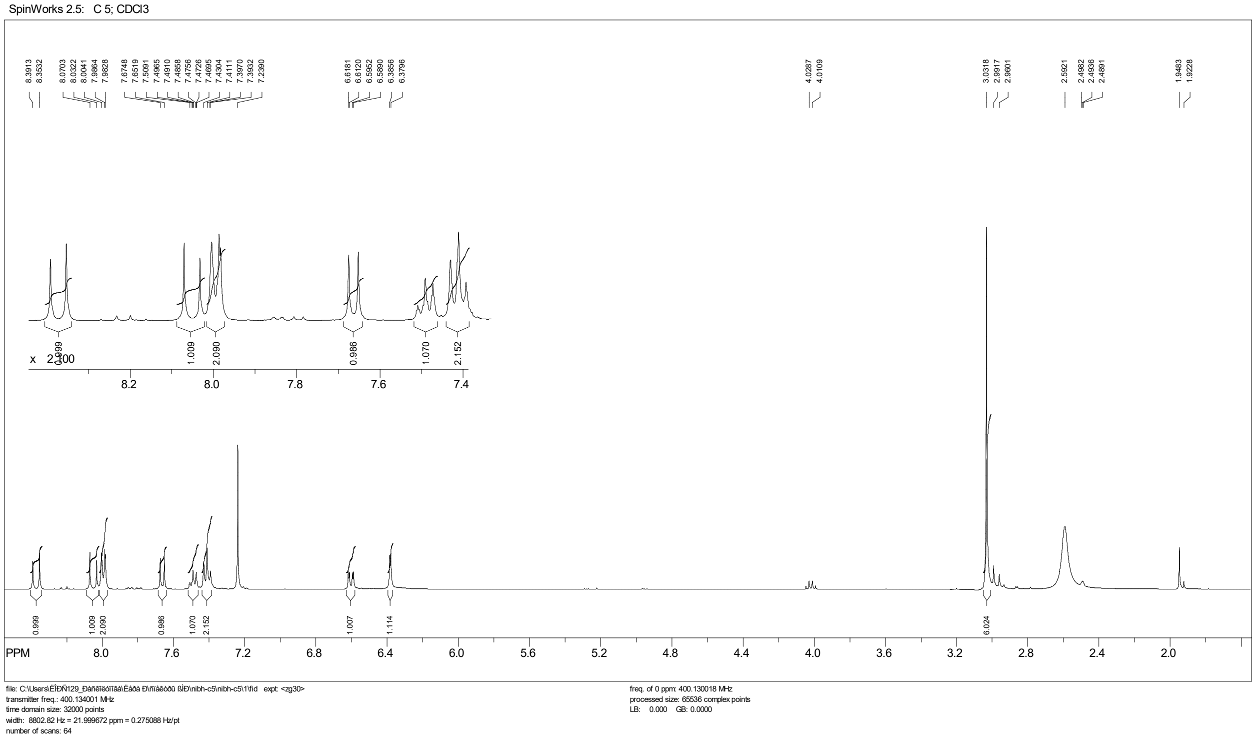

2-Benzoyl-7-(dimethylamino)chromeno[4,3-b]pyrrol-4(1H)-one (7)

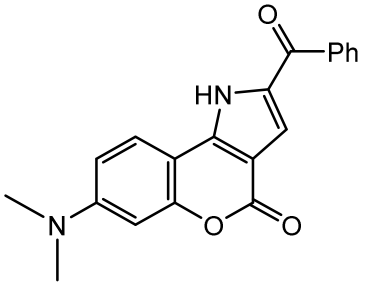

To a suspension of compound (**5**) (0.654 g, 1.9 mmol) in acetone (50 mL) sodium azide (0.200 g, 3.0 mmol) was added and the reaction mixture was vigorously stirred at 40°C for 2 h. Solvent was removed under reduced pressure and the residue was treated with water to give precipitate. Crystals were filtered off and dried in vacuum to give mixture of coumarins **6** and **7**. This solid mixture was suspended in dry toluene (15 mL) and refluxed for 2 h. Reaction mixture was cooled, the precipitate was filtered off, washed with toluene and dried to give pure title compound **7**. Light yellow powder. Yield: 0.554 g, 1.7 mmol, 88%. ^{1}H NMR (DMSO-*d6*): 8.25 (1H, d, *J* 8.9, H9), 7.90 (2H, d, *J* 7.0, *o*-Ph), 7.68 (1H, t, *J* 7.4, *p*-Ph), 7.58 (2H, t, *J* 7.4, *m*-Ph), 7.19 (1H, d, *J* 1.6, H3), 6.80 (1H, dd, *J* 2.5, 9.1, H8), 6.63 (1H, d, *J* 2.4, H6), 3.02 (6H, s, $N(CH_3)_2$); ^{13}C NMR (DCOOD): 183.43, 162.17, 156.41, 148.64, 139.35, 134.46, 132.15, 130.62, 129.83, 125.39, 124.86, 121.41, 114.86, 113.50, 110.65, 106.69, 43.29; MS ESI (m/z): $[M+H]^+$ calcd for $C_{20}H_{17}N_2O_3$ 333.12; found 333.00.

^{1}H NMR

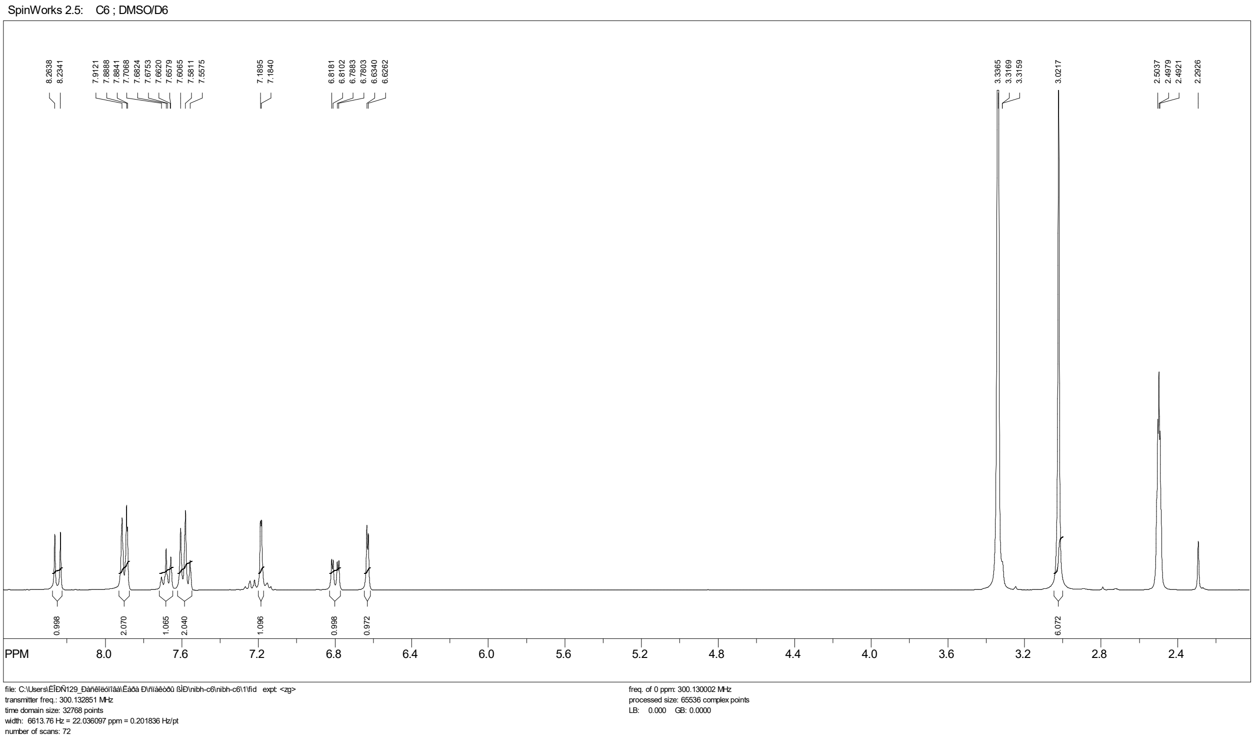

^{13}C NMR

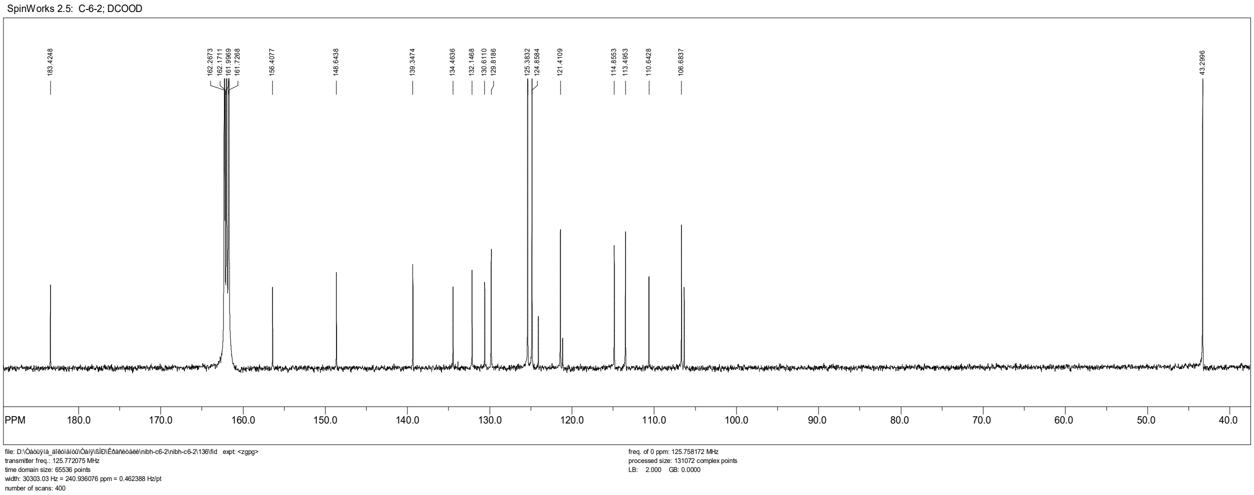

ESI mass spectra

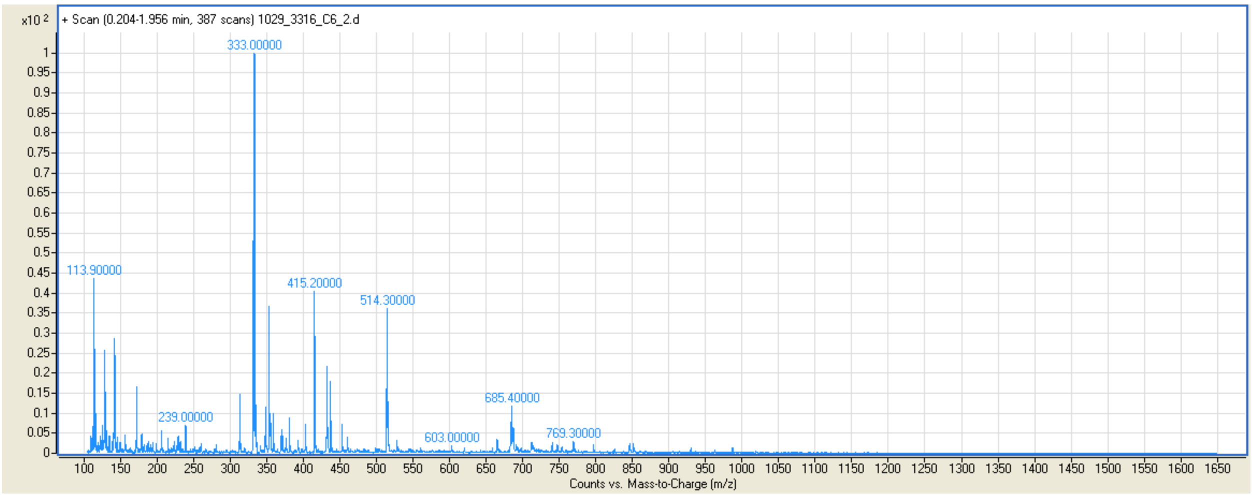

4-(4-Methoxy-4-oxobutyl)-3,5-dimethyl-1H-pyrrole-2-carboxylic acid (10)

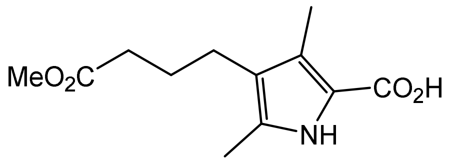

^{1}H NMR (DMSO-*d6*): 10.94 (1H, s, NH), 3.56 (3H, s, OCH_3), 2.28 (2H, t, *J* 7.6, CH_2), 2.26 (2H, t, *J* 7.3, CH_2), 2.14 (3H, s, CH_3), 2.08 (3H, s, CH_3), 1.64-1.54 (2H, m, CH_2).

^{1}H NMR

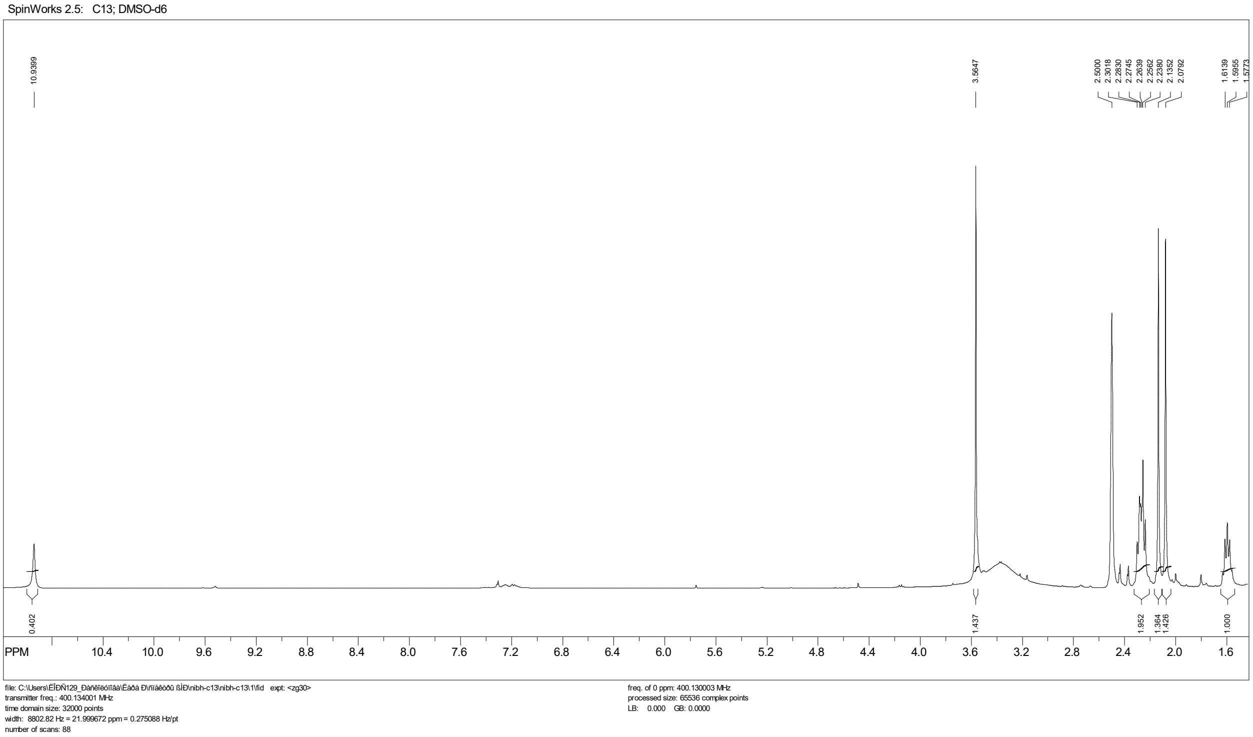

BODIPY compound (8)

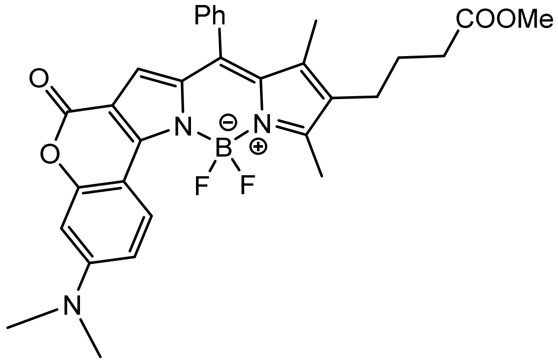

^{1}H NMR

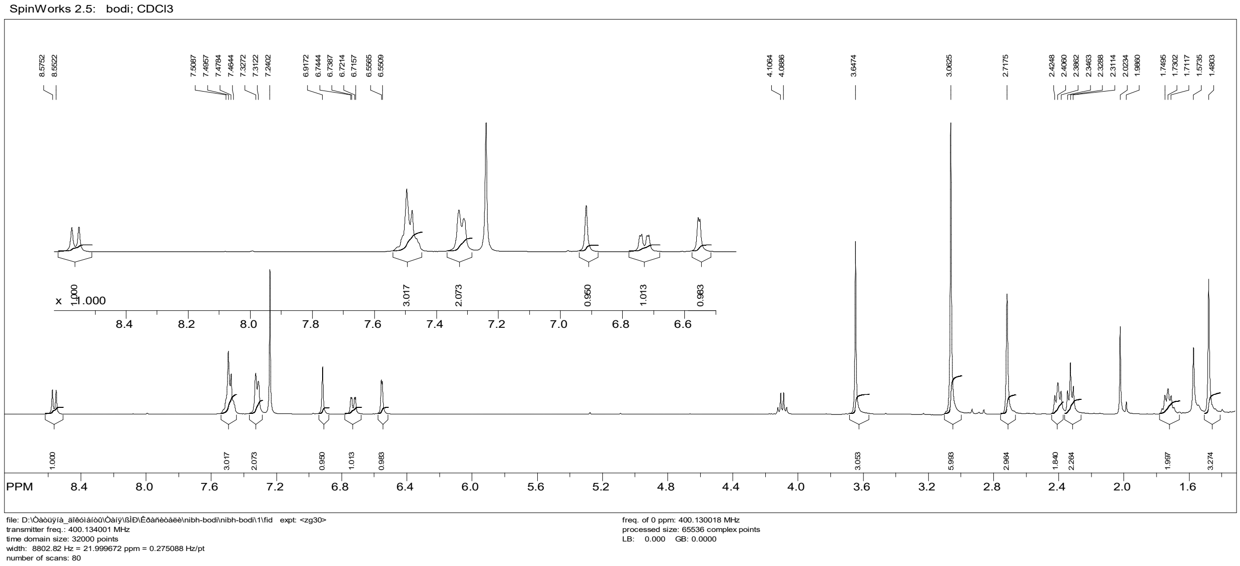

^{13}C NMR

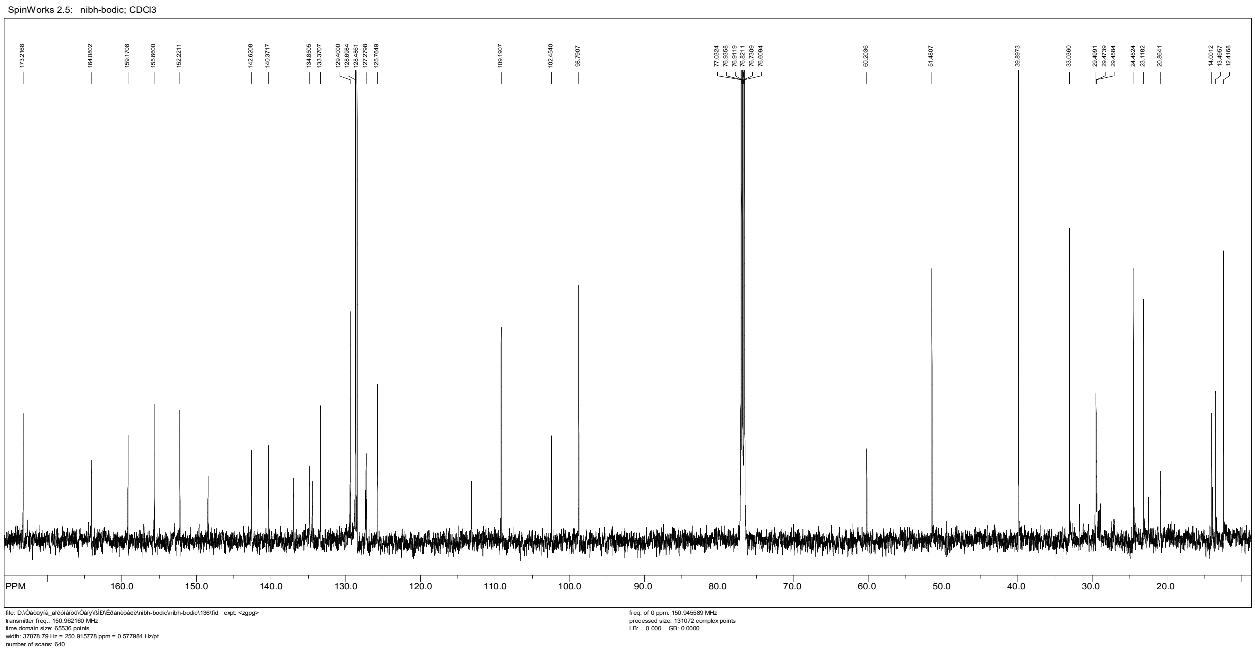

ESI mass spectra

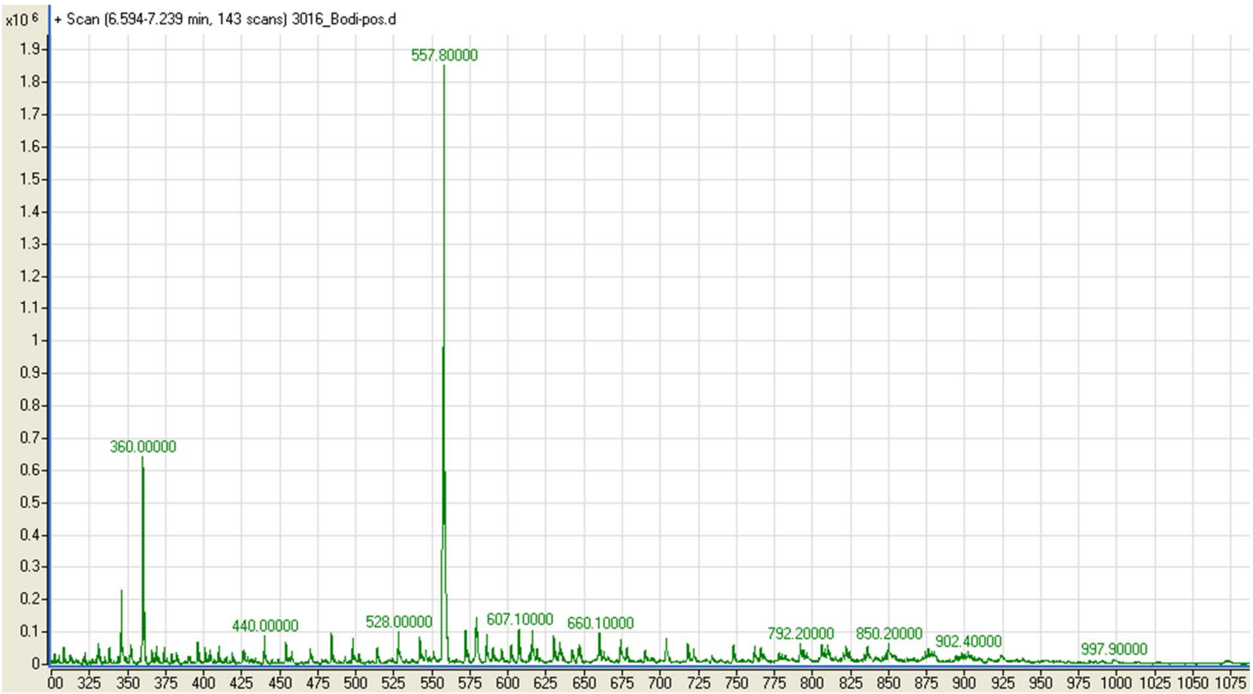
+ Scan (6.594-7.239 min, 143 scans) 3016_Bodi-pos.d
x10 6
557.80000
360.00000
440.00000
528.00000
607.10000
660.10000
792.20000
850.20000
902.40000
997.90000

BODIPY-COOH (1)

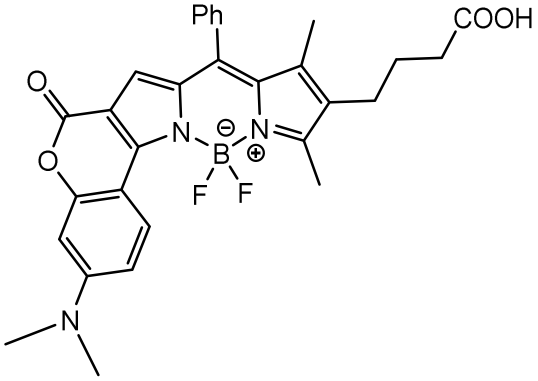

^{1}H NMR

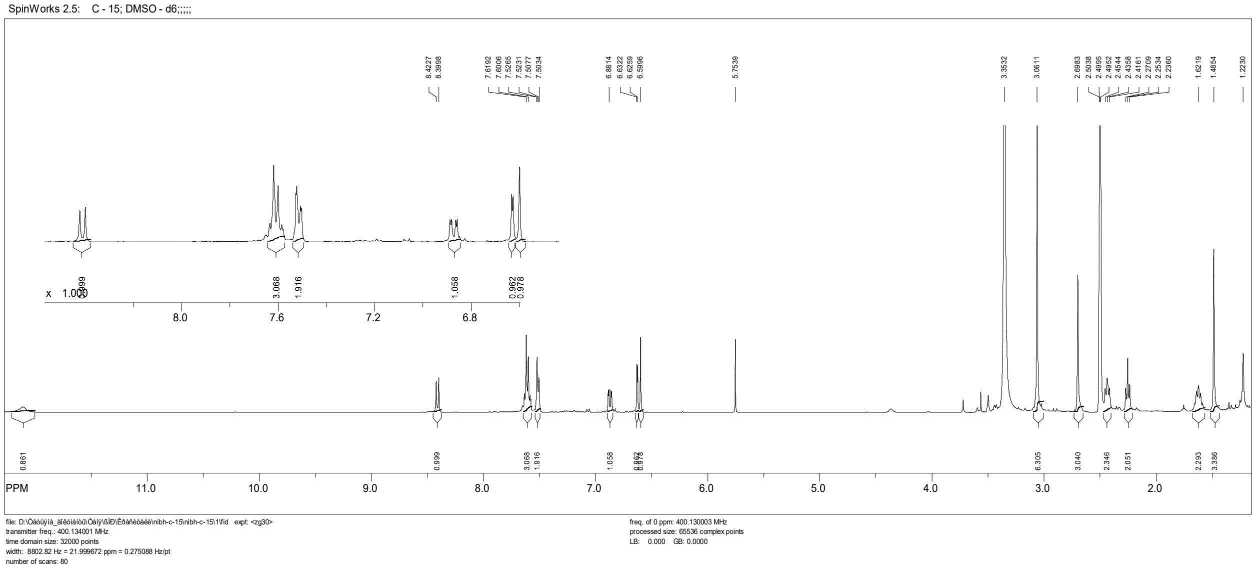

^{13}C NMR

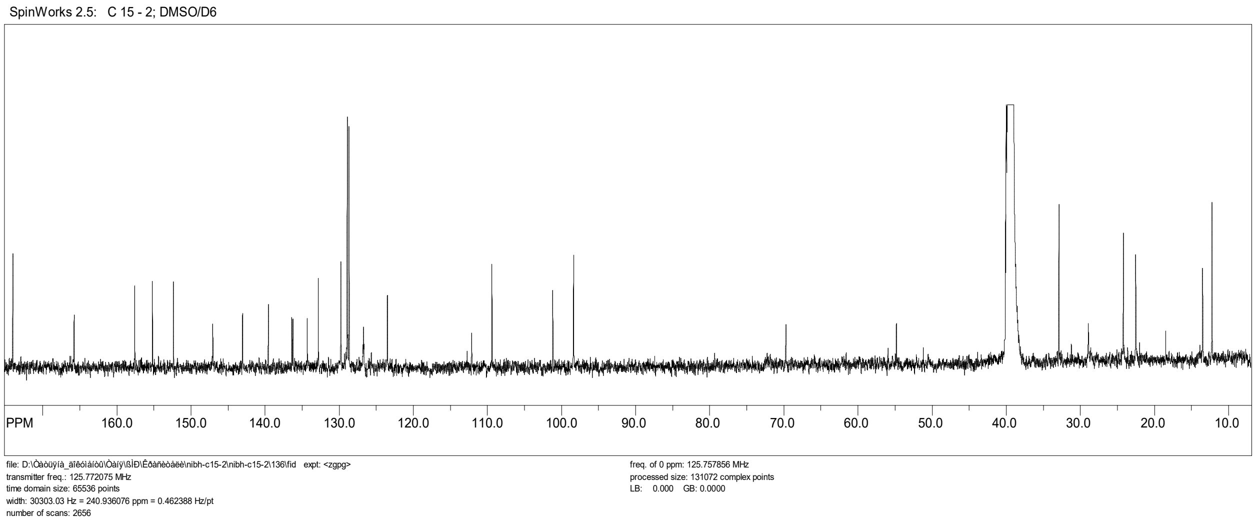

ESI mass spectra

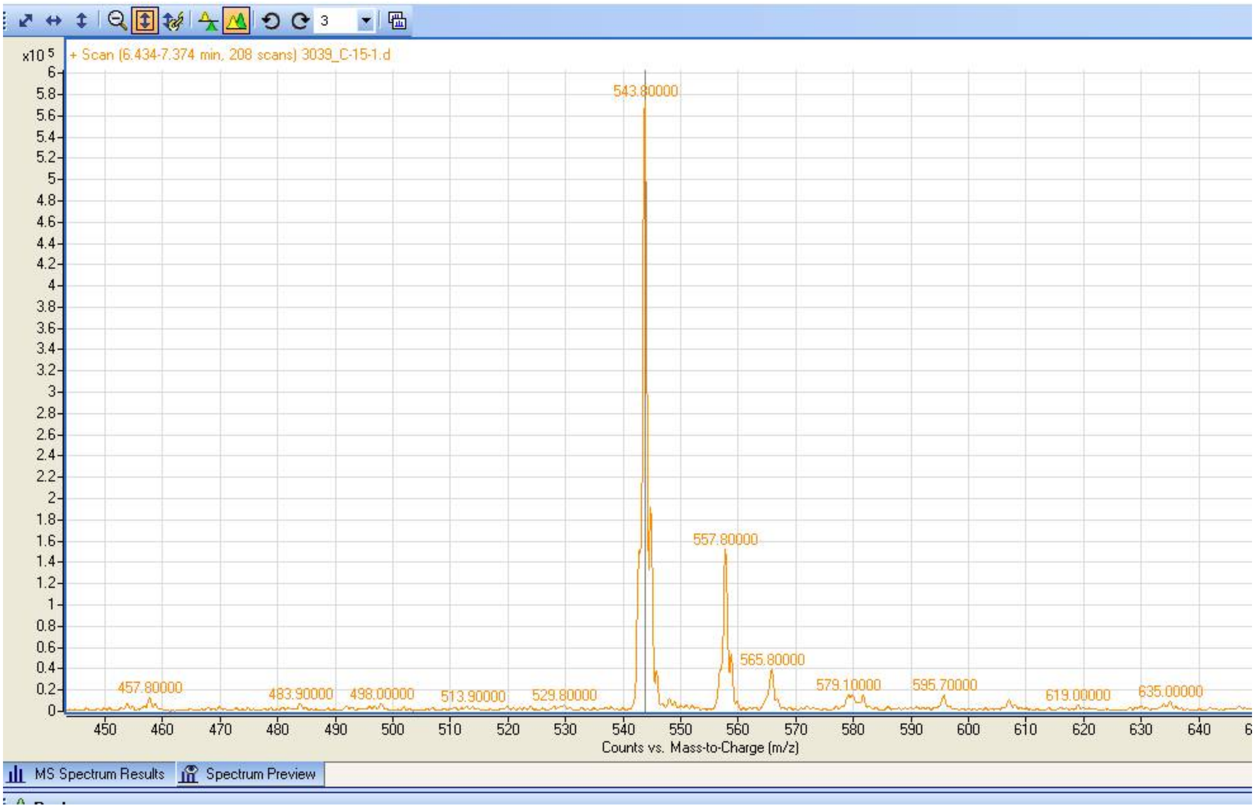

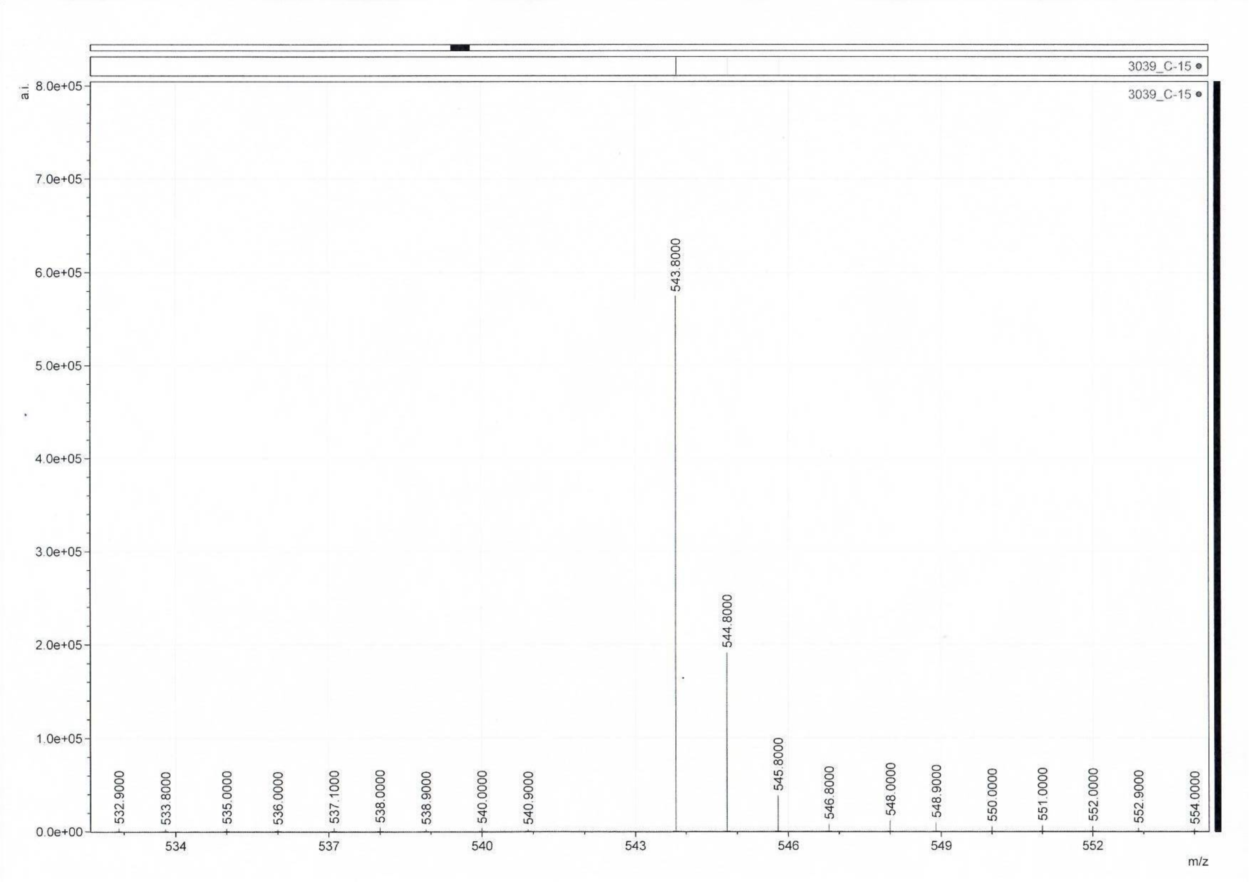

BODIPY-COOH (11)

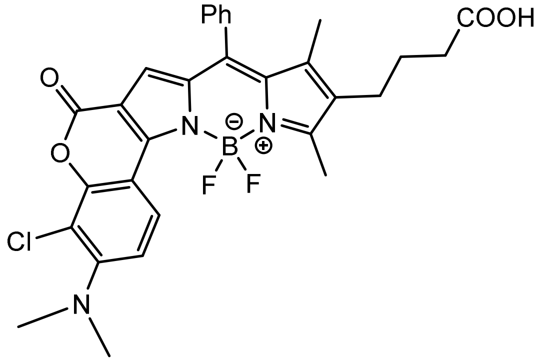

^{1}H NMR

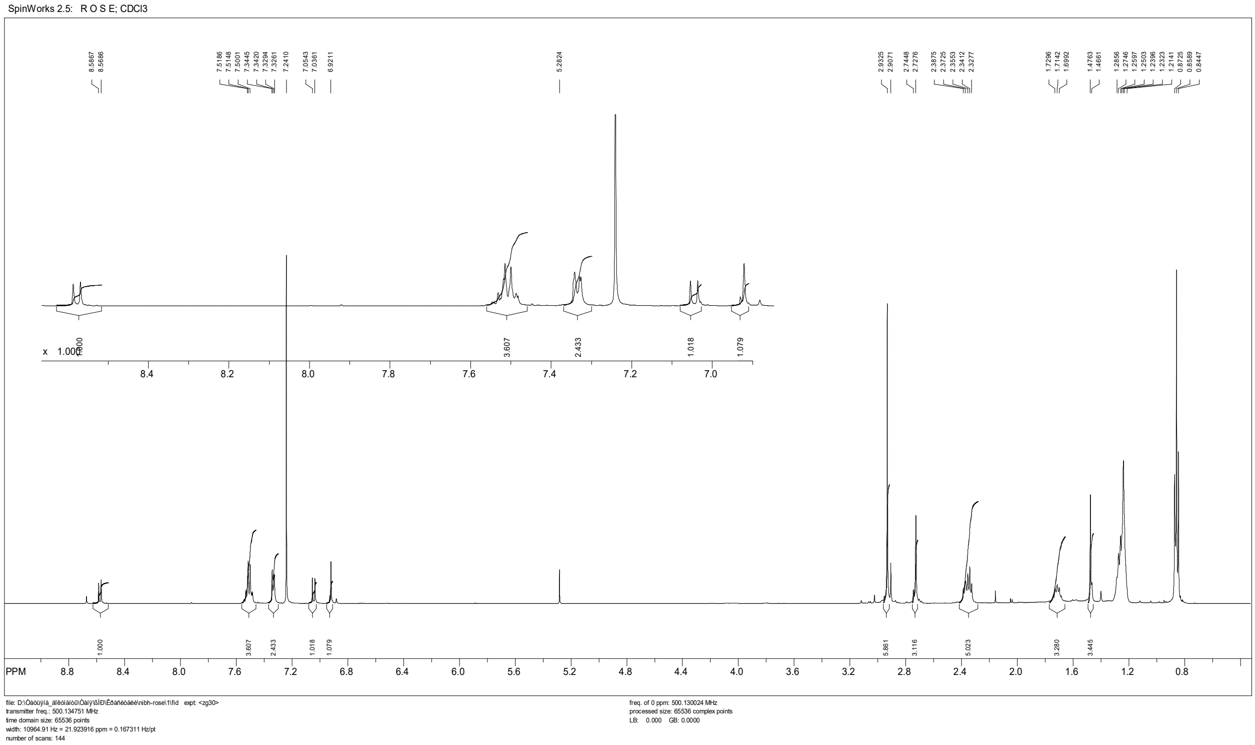

^{13}C NMR

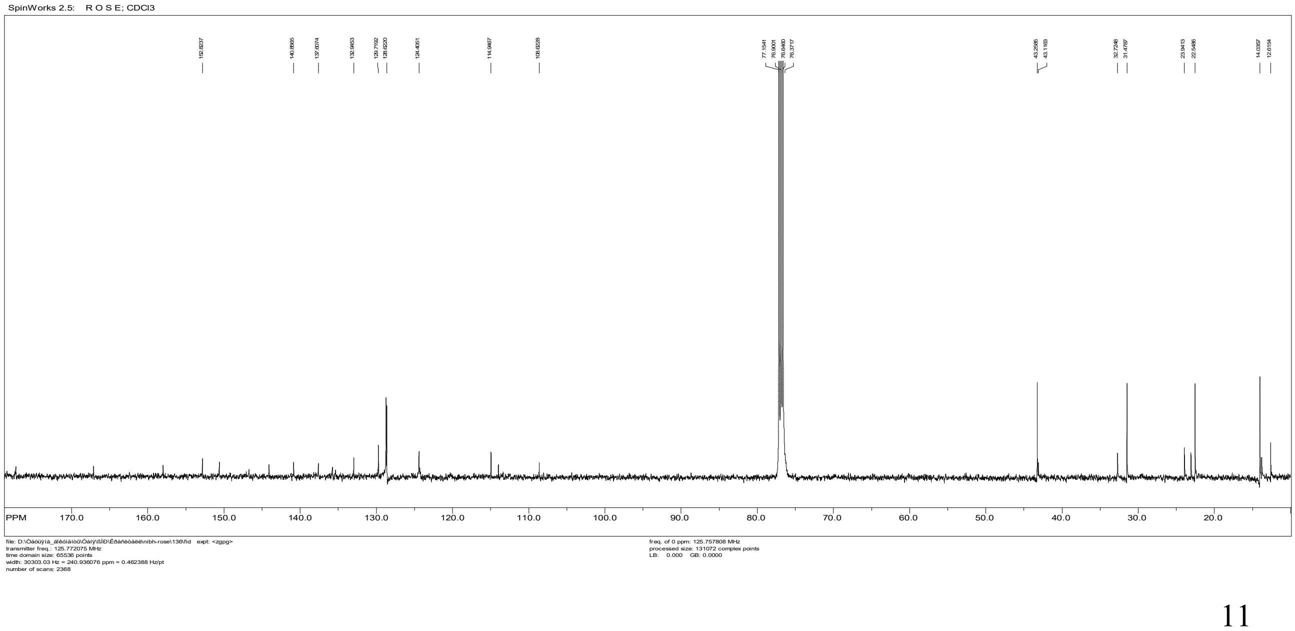

ESI mass spectra

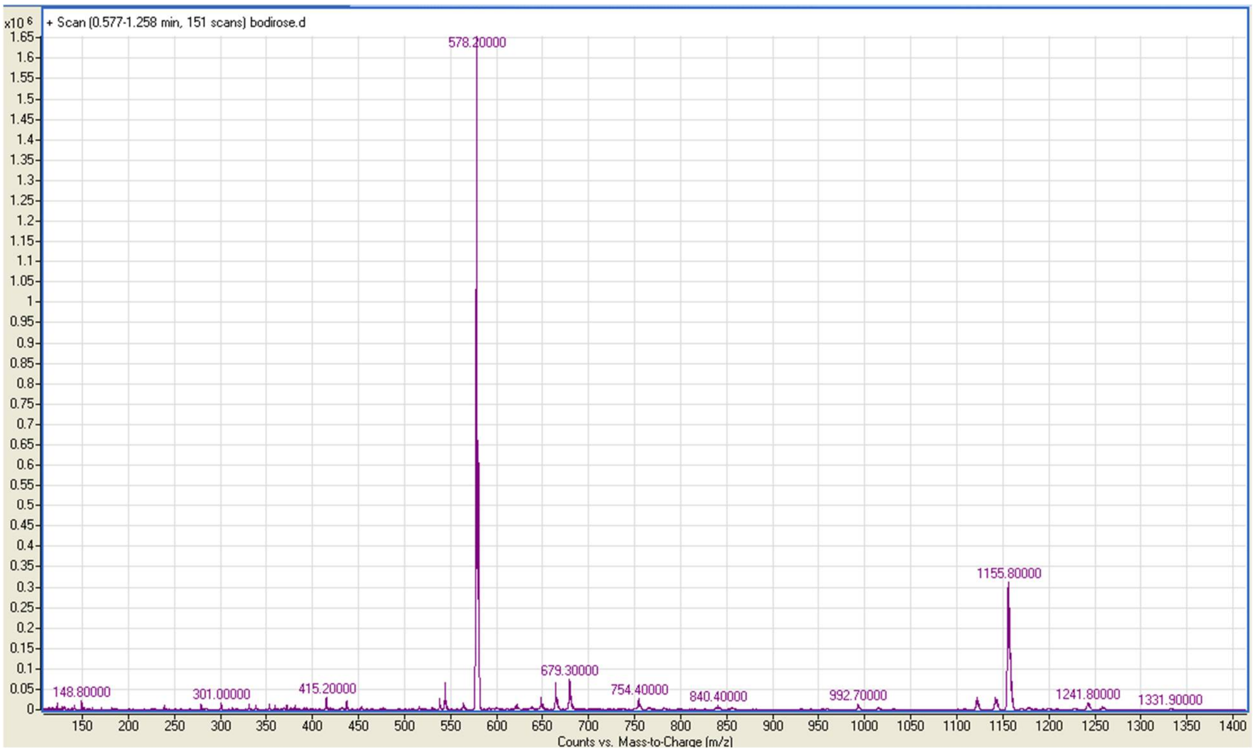

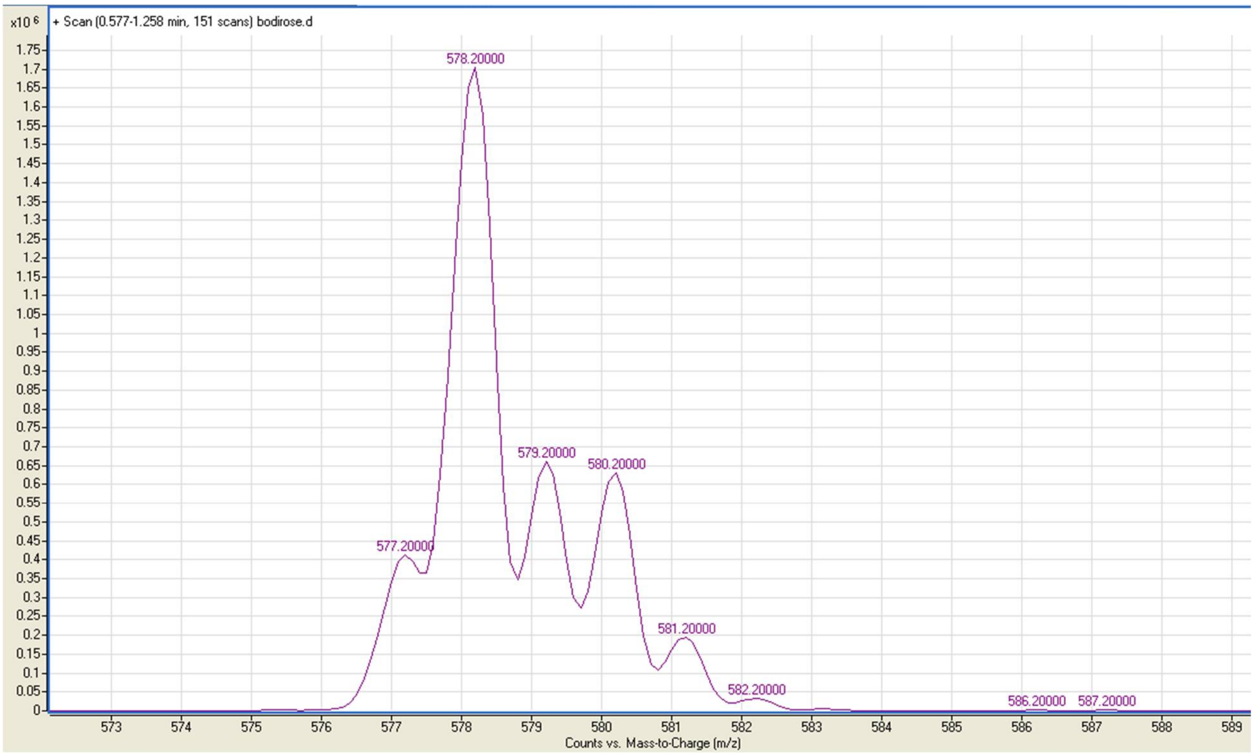

BODIPY maleimide derivative (12)

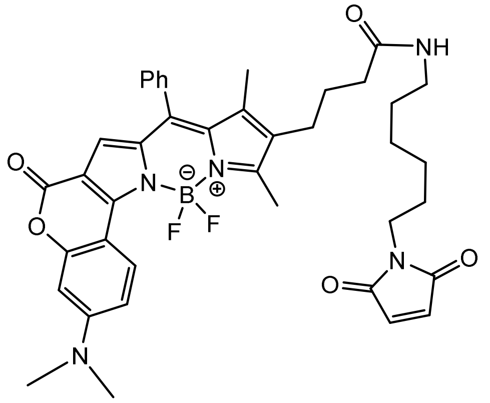

^{1}H NMR

SpinWorks 2.5: BODIM; CDCL3

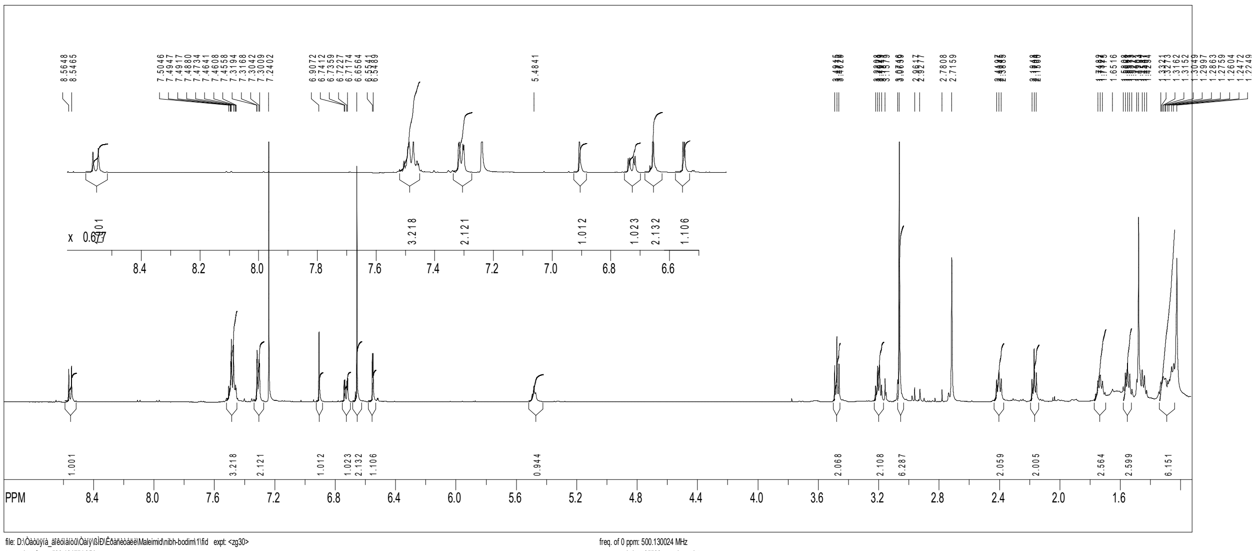

file: D:\Ôàéëû\ä_ãëö\ä\öö\Ôàéÿ\ßÎÐ\Êðàñèòåëè\Maleimid\nibh-bodim\1\fid expt: <zg30>
transmitter freq.: 500.134751 MHz
time domain size: 65536 points
width: 10964.91 Hz = 21.923916 ppm = 0.167311 Hz/pt
number of scans: 64

freq. of 0 ppm: 500.130024 MHz
processed size: 65536 complex points
LB: 0.000 GB: 0.0000

^{13}C NMR

SpinWorks 2.5: BODIM; CDCL3

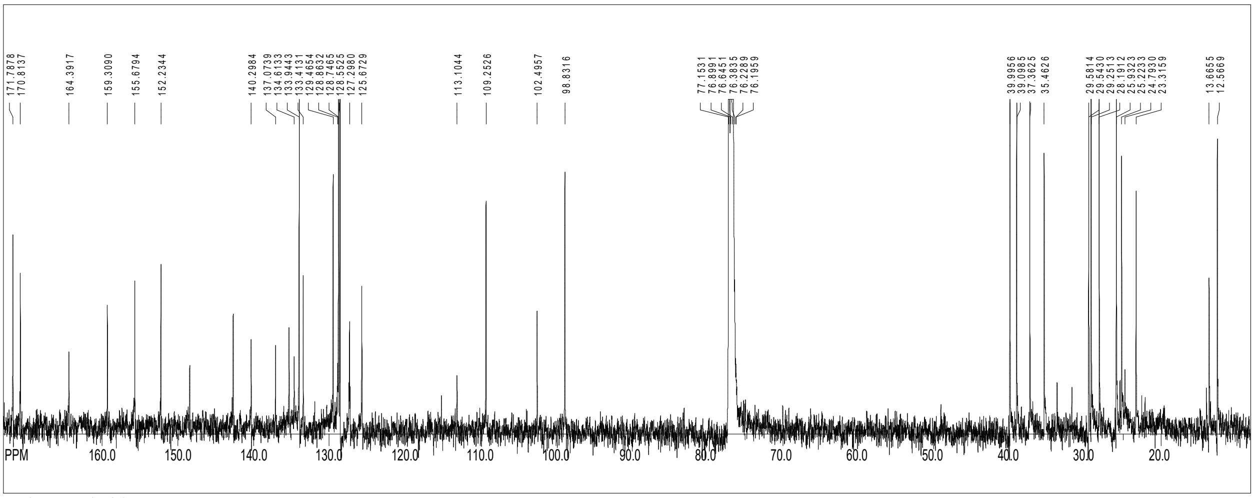

file: D:\Ôàéëû\ä_ãëö\ä\öö\Ôàéÿ\ßÎÐ\Êðàñèòåëè\Maleimid\nibh-bodim\136\fid expt: <zgpg>
transmitter freq.: 125.772075 MHz
time domain size: 65536 points
width: 30303.03 Hz = 240.936076 ppm = 0.462388 Hz/pt
number of scans: 2608

freq. of 0 ppm: 125.757808 MHz
processed size: 131072 complex points
LB: 0.000 GB: 0.0000

^{19}F NMR

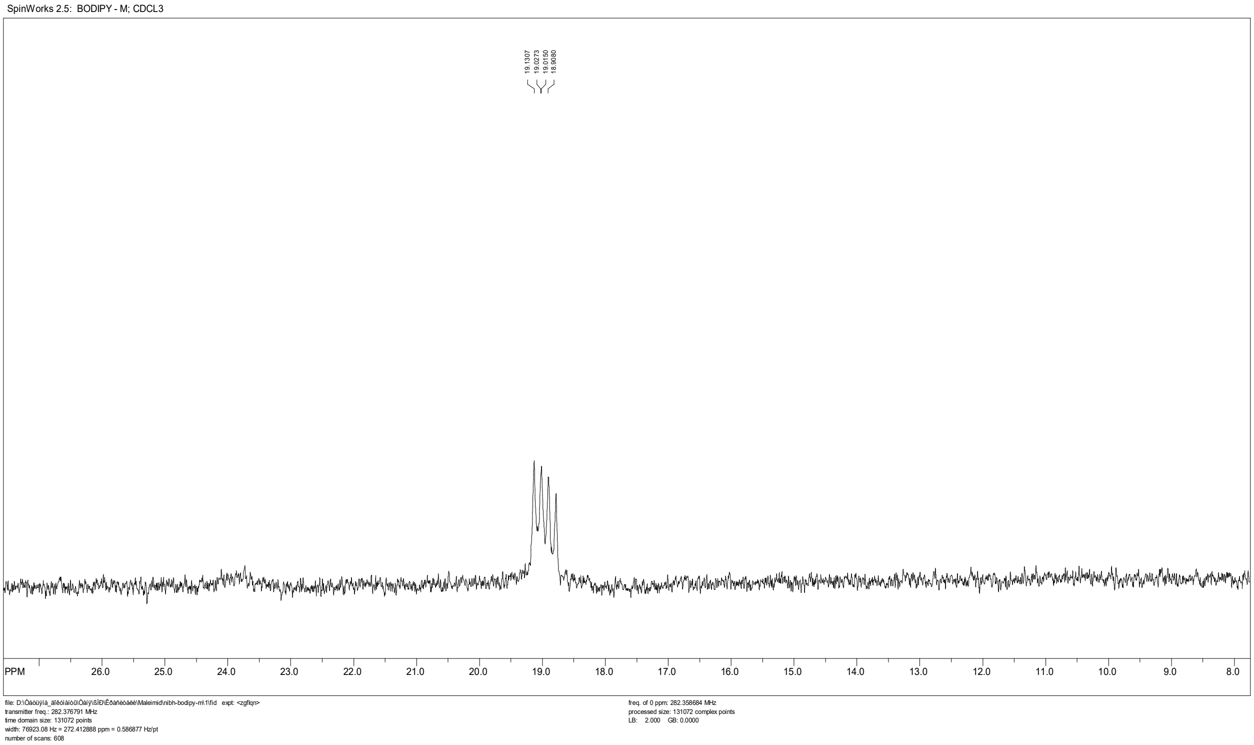

ESI mass spectra

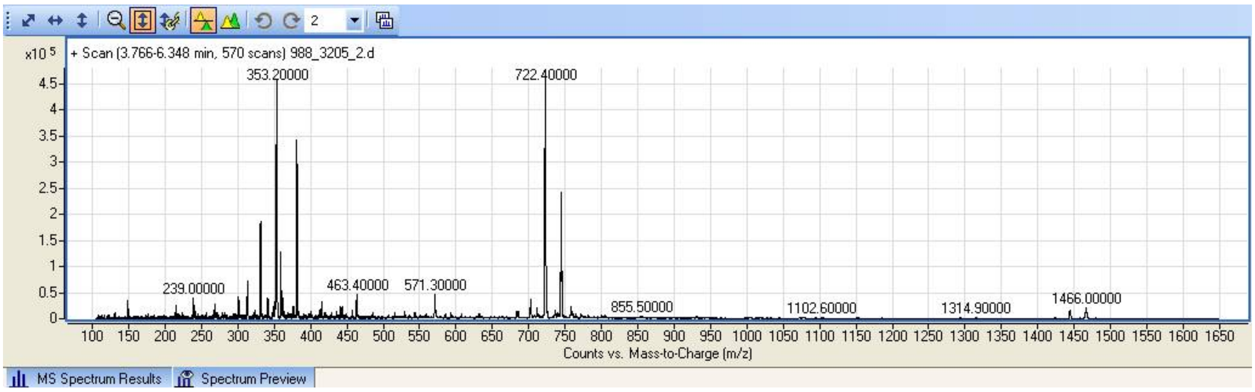

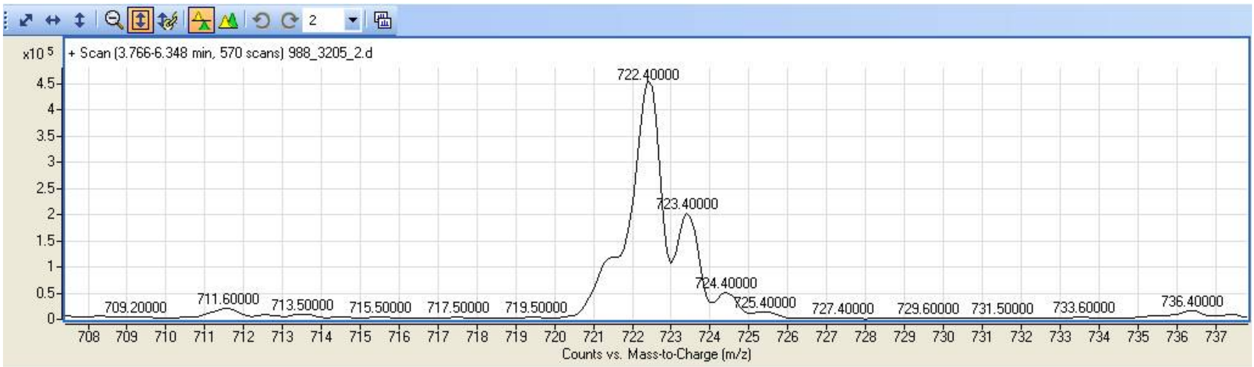

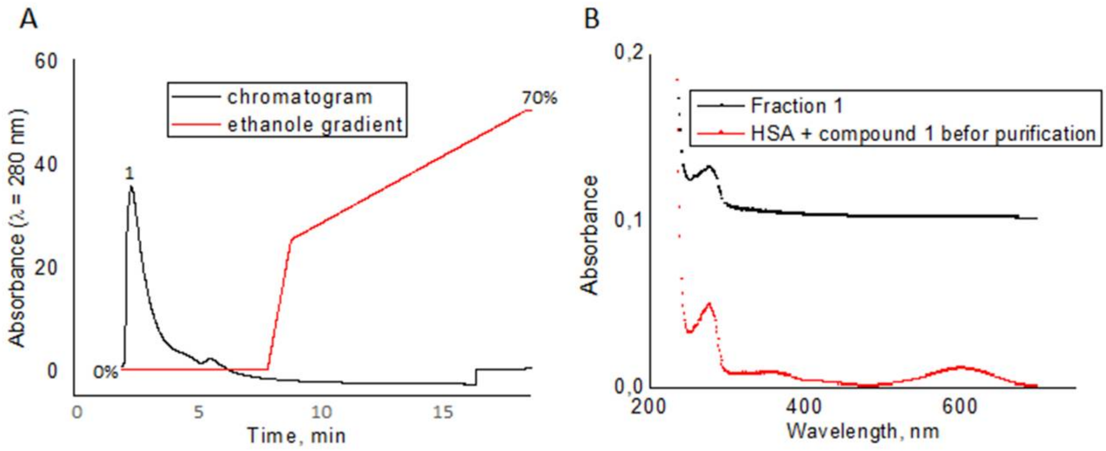

Figure S1. Panel A: ion exchange HPLC of a mixture of HSA and BODIPY dye (**1**). Polysil CA-500 column, 10 µm, 250 x 4.6 mm., elution: 0.3 M phosphate buffer, pH 7.4, ethanol gradient 0-70%, UV detection at 280 nm; panel B: UV-vis spectra of the HSA and BODIPY (**1**) mixture (red line) and fraction 1 (black line).
